# Kombucha Beverages Produced from Fruits, Vegetables, and Plants: A Review on Their Pharmacological Activities and Health Benefits

**DOI:** 10.3390/foods12091818

**Published:** 2023-04-27

**Authors:** Natthinee Anantachoke, Ratchanee Duangrat, Tanyarat Sutthiphatkul, Duangjai Ochaikul, Supachoke Mangmool

**Affiliations:** 1 Department of Pharmacognosy, Faculty of Pharmacy, Mahidol University, Bangkok 10400, Thailand; 2 Department of Pharmacology, Faculty of Science, Mahidol University, Bangkok 10400, Thailand; 3 Department of Biology, School of Science, King Mongkut’s Institute of Technology Ladkrabang, Bangkok 10520, Thailand

**Keywords:** kombucha tea, kombucha beverage, biological activity, pharmacological effect, toxicity

## Abstract

Kombucha is a traditional health beverage produced by fermenting sweetened tea with a symbiotic culture of bacteria and yeasts. Consumption of kombucha beverages has been growing and there is kombucha commercially available worldwide as one of the most famous low-alcohol beverages. Kombucha beverages have been claimed to have beneficial effects on human health because they contain a variety of bioactive compounds that possess various functional properties. At present, several kinds of raw material (e.g., milk, fruit, vegetables, and herbs) have been fermented with kombucha consortium and consumed as kombucha beverages. Although several studies have been written regarding the biological activities of kombucha and raw materials, there is however little information available on the characterization of their components as well as the biological activities of fermented kombucha from many raw material mixtures. Several pharmacological activities were reviewed in the scientific literature, describing their potential implications for human health. In addition, the adverse effects and toxicity of kombucha consumption were also reviewed. In this study, we focused on the main and latest studies of the pharmacological effects of kombucha beverages produced from various kinds of raw materials, including antioxidant, anti-inflammatory, immunomodulatory, antimicrobial, anticancer, antidiabetic, antihypertensive, and antihyperlipidemic effects in in vitro and in vivo studies.

## 1. Introduction

Kombucha is a traditional beverage manufactured from fermenting tea (*Camellia sinensis* (L.) Kuntze) with a symbiotic culture of bacteria and yeasts (SCOBY) in a sweet medium under aerobic conditions for several days. The SCOBY is mainly integrated by mixing various types of yeasts and acetic acid bacteria (AAB) that produce a cellulose biofilm layer, called tea fungus, found on the surface of the growth medium [1,2]. During the fermentation process, several components are produced and contained in the kombucha, including organic acids, sugars, water-soluble vitamins, amino acids, lipids, proteins, hydrolytic enzymes, ethanol, polyphenols, minerals, and others [3,4,5,6]. These bioactive compounds are mainly found in the liquid growth medium during the course of fermentation [4,5].

Currently, kombucha consumption has been growing and is commercially available worldwide as one of the most popular beverages because of claims regarding their nutritional properties and the potential health benefits of the beverage. In addition to tea (*C. sinensis*), the uses of many raw materials (e.g., fruit, vegetables, milk, coffee, soy, and herbs) for fermentation with kombucha consortium have been reported and could be produced as kombucha beverages. In addition, many metabolic reactions occur during the fermentation process, leading to the formation of various metabolites [7,8,9]. After fermentation with kombucha consortium, the chemical components contained in the raw materials could be metabolized into other compounds by both bacteria and yeasts during fermentation [5,6,9]. Therefore, the components found in kombucha beverages depend on the types of raw materials and kombucha consortium, especially the species of regional bacteria and yeasts that can produce the differences in components and nutritional properties.

Previous studies have demonstrated the beneficial effects of kombucha beverages; however, there is little information on their pharmacological activities or the characterization of their bioactive components from many raw material mixtures. At present, the food studies that ensure safety and contribute to health promotion regarding the development and consumption of kombucha beverages have increased in frequency in recent years [1,10,11]. In this review, we will discuss the biological activities of various kinds of raw materials fermented with kombucha consortium, including antioxidant, anti-inflammatory, and antimicrobial activities as well as antidiabetic, antihypertensive, anticancer, and antihyperlipidemic effects. Intensive research on the pharmacological effects of kombucha fermented with several raw materials is an essential tool to gain a better understanding of the mechanisms of these protective properties in the physiological activities of kombucha beverages.

We aimed to gather data from many scientific studies on kombucha beverages prepared from a variety of raw materials and focused extensively on their potential pharmacological properties in in vitro and in vivo studies, improving knowledge about the nutritional properties of these health beverages. Even though the effects of kombucha beverages have been studied in animal models for many conditions, the adverse effects and toxicity of kombucha in animal and human bodies remain unclear. Therefore, we also discussed the adverse effects of kombucha beverages.

## 2. Kombucha

Kombucha, a fermented beverage, was originally consumed in Asia and is made on a large scale commercially as well as in households and small businesses all over the world. Due to the potential health benefits of kombucha beverages, their consumption has been growing worldwide. At present, a variety of kombucha beverages are sold in markets all over the world [1,12]. Kombucha is a useful non-alcoholic or low-alcohol beverage that has slightly sweet and sour flavors. Depending on where you reside, kombucha is known by many various names, including “fungus tea”, “Kargosok tea”, “Indian fungus tea”, “Manchu fungus”, “teakwass”, and many others [4]. Kombucha has seen the fastest increase in the health beverage market among other fermented beverages. Furthermore, it is widely available on numerous websites and sold in retail food stores worldwide in a variety of flavors.

Many raw materials (e.g., fruit, vegetables, milk, and herbs) can be added and fermented with a SCOBY (or kombucha formula) and the resulting liquid media are consumed as kombucha beverages. These beverages have been shown to possess beneficial effects on health because they contain several bioactive compounds that possess many pharmacological properties [13,14,15,16]. These bioactive compounds are derived from the components of kombucha consortium and the metabolite products generated during fermentation as well as the type of raw materials [13,14,15,16].

### 2.1. Symbiotic Culture of Bacteria and Yeast (SCOBY)

**Figure 1:**
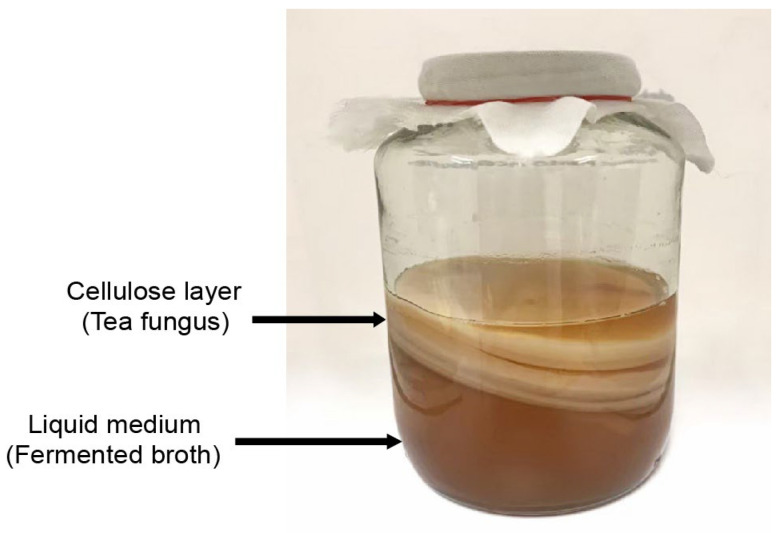
Kombucha prepared using sweet tea after 21 days of fermentation. The fermentation of kombucha resulted in the formation of two main portions: a floating cellulose layer (tea fungus) and the liquid medium (fermented broth).

In the fermentation process, the most abundant prokaryotes in the kombucha starter are the bacteria and yeasts that make up a culture known as a SCOBY. The bacteria and yeasts used for the fermenting of kombucha have variable microbiological composition depending on the region of the country, the raw materials used, and the conditions of the fermentation process [6,17,18]. After 21 days of fermentation, a SCOBY can produce two main portions [7]: a floating cellulose layer which is called tea fungus and the liquid medium which called fermented broth (Figure 1). The sour liquid medium contains a variety of components, including organic acids, amino acids, ethanol, vitamins, minerals, enzymes, and bioactive compounds [4,13]. Several yeast genera (*Saccharomyces*, *Zygosaccharomyces*, *Dekkera*, and *Candida)* and acetic acid bacteria (*Acetobacter*, *Gluconacetobacter*, *Komagataibacter*, and *Gluconobacter*) have commonly appeared in starter cultures [4,19]. Additionally, lactic acid bacteria (LAB) such as *Lactococcus* and *Lactobacillus* may also be present in kombucha [3,9,18]. The microbial community in kombucha depends on the conditions of fermentation (e.g., duration, temperature, and selected starter) [6] as well as the raw materials utilized. Therefore, there is no standardization in kombucha’s microbial composition. However, some identical species and strains of bacteria and yeasts are commonly found in kombucha consortia.

#### 2.1.1. Bacteria

AAB, which are the predominant aerobic bacteria found in kombucha cultures, have the ability to oxidize glucose into gluconic acid and glucuronic acid and can also use ethanol as a substrate to produce acetic acid [3,4]. The production of ethanol and acetic acid can inhibit the growth of pathogenic bacteria in kombucha [20]. Some dominant bacteria, including *Acetobacter xylinum* and *Komagataeibacter* spp., utilize glucose to produce bacterial cellulose. At the same time, on the surface of the fermenting liquid, bacteria produce a floating cellulose network known as “tea fungus”. The network is one of the distinctive characteristics of the culture in addition to being a secondary metabolite of kombucha fermentation [4]. AAB are also responsible for producing acetic acid and bacterial cellulose, as well as glucuronic acid [21,22]. LAB can use glucose either via the pentose phosphate pathway, which produces lactic acid, ethanol, and carbon dioxide, or the Embden-Meyerhof-Parnas pathway, where lactic acid is the primary metabolite [6].

#### 2.1.2. Yeast

*Saccharomyces* (*Saccharomyces cerevisiae*) and non-*Saccharomyces* (*Zygosaccharomyces* spp., *Shizosaccharomyces* spp., *Dekkera* spp., *Brettanomyces* spp.) yeast strains can be found in kombucha [18]. *Saccharomyces* yeasts are commonly found in industrial settings such as ethanol and alcohol beverage manufacturing [7,22,23]. However, non-*Saccharomyces* yeasts are used as aromatics and to enhance the complexity of final products [3]. The interaction in the fermentation process is initiated by yeasts that hydrolyze sucrose into glucose and fructose by invertase activity and ultimately produce ethanol via glycolysis [17].

### 2.2. Fermentation Process

In general, the typical production of kombucha is based on the types of tea used in kombucha fermentation, such as black tea (post-fermented), oolong tea (semi-fermented), or green tea (non-fermented). Several factors such as types and amounts of raw material, starter culture, sugar content, and fermentation temperature and time can potentially affect the physicochemical characteristics of the obtained kombucha products, including different constituents which consequently affect their biological activities [4,5,6,11]. In addition, the chemical composition of kombucha varies considerably according to the types of tea and raw materials used during the fermentation process. The fermentation of kombucha leads to the formation of two main portions: a floating cellulose layer (tea fungus) and the liquid medium called fermented broth (Figure 1). The duration of fermentation is around 3–5 days up to a maximum of 60 days and the temperature of fermentation ranges from 20 to 30 °C, depending on cultural practices and types of materials [3,4,12,24]. To avoid unwanted contamination, it is necessary to use sanitized instruments and work in clean areas during the fermentation process.

### 2.3. Substances and Biological Components Found in Kombucha Beverages

**Figure 2:**
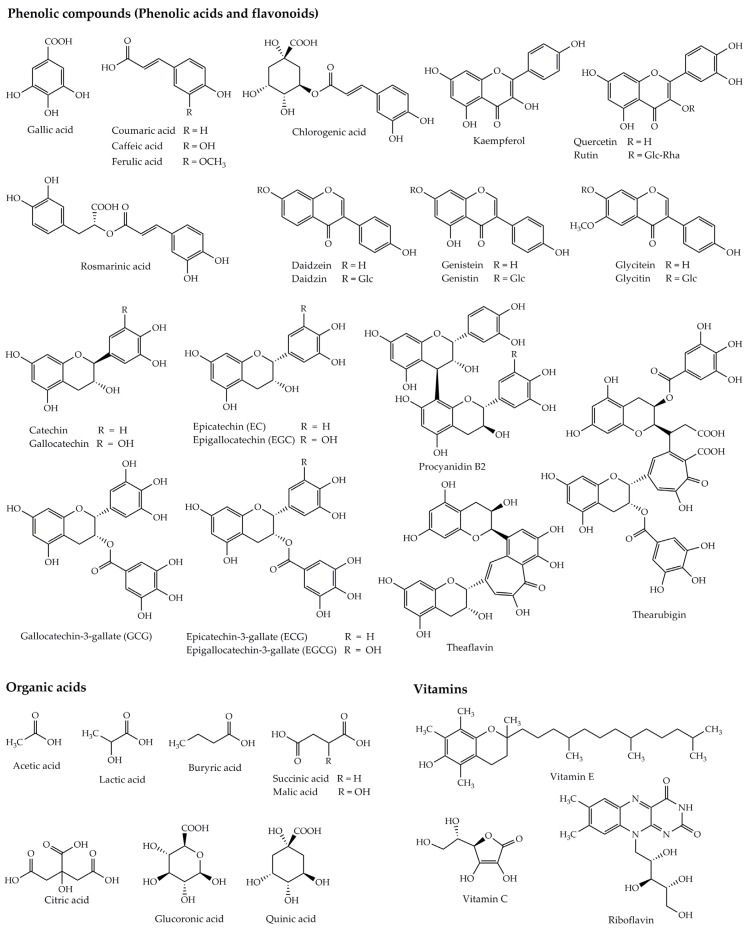
Chemical structure of some substances found in kombucha tea beverages.

Kombucha beverages contain a variety of substances and biological components (Figure 2) which come from tea (substrate) and microorganisms as well as other raw materials used during fermentation [6]. As already stated, the action of invertase enzyme secreted by yeasts hydrolyzes sugar substrate into its monomers (fructose and glucose) and further converts them to ethanol through glycolysis. In addition, glucose and ethanol utilization by AAB results in the production of glucuronic acid and acetic acid, respectively, indicating that these acids are the main organic acids found in fermented kombucha. Other organic acids such as citric, lactic, malic, tartaric, malonic, succinic, oxalic, and pyruvic acids were also found in kombucha [5,7,8,9,18]. In addition, kombucha is composed of many substances mainly derived from tea, including minerals (potassium, manganese, and fluoride), vitamins (B, C, E, and K) as well as other compounds that are formed as the result of numerous reactions occurring during the fermentation of tea [5,7,8,9,18]. Furthermore, kombucha contains a wide variety of chemical compounds, including polyphenols, sugars (sucrose, glucose, and fructose), ethanol, amino acids, pigments, lipids, proteins, and hydrolytic enzymes [13]. Most of these substances have many pharmacological effects and can be considered bioactive compounds. Collectively, several parameters influence the properties and components of kombucha beverages, including the type of tea, fermentation duration, temperature, and type and contents of SCOBY [1,3,4,11]. We discuss the biological activities of bioactive compounds in Section 3.

### 2.4. Adverse Effects and Toxicities of Kombucha Beverages

The therapeutic benefits of kombucha have been documented in several studies at the cellular level and in organisms. At present, the Food and Drug Administration (FDA) has approved kombucha as safe for human consumption and the recommended dose for dairy drinks provided by the Centers for Disease Control and Prevention (CDC) is 4 ounces (approximately 110 mL). Several studies also supported the safety of kombucha used in animal toxicological models [25,26]. However, it is important to note that there have been reported cases of unexplained severe illness, including death, associated with daily consumption of kombucha over periods of approximately two months [27]. Some individuals experienced undesired symptoms after consuming kombucha, such as shortness of breath, throat tightness, headache, nausea, vomiting, dizziness, neck pain, and jaundice [28]. Similar to other substances, kombucha teas can cause an allergic reaction in the body as demonstrated in the case of one patient [28]. During fermentation, alcohol can be produced in kombucha at levels ranging from 0.7–1.3%. However, even in small amounts, alcohol can cause harmful effects to pregnant and lactating women as well as young children, as they are sensitive to alcohol [29]. Therefore, these populations must avoid kombucha consumption.

The contraindications of kombucha use are of particular concern in individuals with pre-existing health conditions, such as immunosuppressed patients. Within 15 h of kombucha consumption, one HIV patient showed hyperthermia, lactic acidosis, and acute renal failure [30]. An elderly female patient with hypothyroidism and diabetes mellitus (DM) also showed hepatotoxicity after consuming kombucha for a month [31]. In addition, a 54-year-old asthmatic woman was diagnosed with severe metabolic lactic acidosis after drinking kombucha for several months [32]. Given these incidents, it is crucial to be aware of the potential side effects of kombucha, especially for those with sensitive health conditions. The high acidity of kombucha must also be taken into account, as excessive consumption can lead to metabolic acidosis in some individuals [27]. Moreover, kombucha contains various microbes, including bacteria and yeasts, and their effects on human gut microbiota require further evaluation in both healthy and diseased individuals.

The mechanism underlying the toxicity of kombucha is still unclear, but several studies have arisen to shed light on the causes. It is believed that the active components present in kombucha, combined with poor hygiene practices during preparation and potential contamination by the presence of pathogenic microorganisms, may contribute to its adverse effects [8]. Normally, kombucha itself does not contain pathogenic microorganisms because it has a low pH and high acidity [27], as well as a strong symbiosis with the microbiota that can prevent contamination by bacteria [33]. However, homemade kombucha is prone to pathogenic microorganism contamination such as *Aspergillus* spp. due to unhygienic preparation, which can cause toxicity [27,28]. Thus, the hygienic preparation of kombucha requires strict control over equipment such as clean stations and vessels and hygienic SCOBY handing. Moreover, the pasteurization of kombucha or the use of commercial SCOBY culture can also be considered.

Two symptomatic lead poisoning cases were reported in a married couple who consumed kombucha brewed in a ceramic pot for six months. This toxicity was believed to be due to the organic acids formed during fermentation which eluted lead from glazing material on the ceramic vessel [34]. To avoid such incidents, the use of containers made of food-grade materials, such as glass or stainless steel, are recommended for the preparation and storage of kombucha. Furthermore, the pH of kombucha must be closely monitored as it decreases over time. A pH below 2.5 results in an overproduction of acetic acid, causing undue acidity, which can be harmful to the body. Therefore, pH during fermentation must be tightly controlled [12]. Finally, as kombucha recipes may vary across regions, it is advised to follow the FDA Model Food Code guidelines in order to ensure the safe production of kombucha [35].

## 3. Pharmacological Activities of Kombucha Beverages

### 3.1. Antioxidant Activities

At present, the aging population is increasing worldwide. Many elderly people are suffering from multimorbidity, of which the prevalence increases with age [36]. Many chronic diseases, especially metabolic disorders, cardiovascular disease, neurodegenerative diseases, musculoskeletal diseases, and cancer, are common causes of multimorbidity [37] and associated with various factors such as genetic variation, lifestyle, nutritional, living environment, and socioeconomic factors [36,38]. Chronic diseases and aging are mainly caused by physiological function impairments and homeostatic imbalance which involves oxidative stress [38,39,40]. Reactive oxygen species (ROSs) and reactive nitrogen species (RNSs), including superoxide, hydroxyl, and nitric oxide (NO) radicals can be naturally generated in our bodies by various biological and pathophysiological processes. these intrinsic ROSs play an important role in several physiological and cellular signaling processes especially for the immune system. A small amount of ROSs and RNSs are required for microbial killing in phagocytosis as well as the regulation of many cellular functions and homeostasis [39,40]. However, excessive ROS production from both intrinsic and exogenous sources can cause oxidative damages to proteins, lipids, DNA, cells, and tissues, leading to cellular dysfunction and cell death [39,40,41]. Normally, there are endogenous antioxidant defense mechanisms for reducing high ROS levels and overproduction. These cellular antioxidant enzymes, including catalase (CAT), glutathione peroxidase (GPx), glutathione reductases (GR), glutathione-S-transferase (GST), superoxide dismutase (SOD), and glucose-6-phosphate dehydrogenase are protein catalysts involved in the detoxification of free radicals and the inhibition of ROS formation in the cells [39,42]. Apart from enzymatic antioxidants, endogenous non-enzymatic antioxidants such as glutathione, alpha-lipoic acid, thioredoxin, melatonin, and coenzyme Q play an important role in free radical-scavenging, regeneration of oxidized antioxidants, and the protection of some vital biomolecules from oxidative damages. Consequently, antioxidant deficiency or excessive ROS and RNS production can disrupt cellular redox homeostasis and leave the cells vulnerable to oxidative stress [39]. In order to protect and restore cells from the oxidative damages, exogenous antioxidants such as phenolic compounds, carotenoids, and vitamins (A, C, and E) are potential free radical scavengers and reducing agents which can alleviate the toxicity of the free radicals through an oxidation–reduction reaction [39,40,43]. The main natural sources of exogenous antioxidants are medicinal plants, vegetables, fruit, cereals, spices, edible flowers, mushrooms, etc. The consumption of antioxidant-rich foods and beverages, as well as food supplements, is beneficial for health promotion and oxidative stress-related disease prevention [40,43].

Tea (*C. sinensis*) is well-known as a good source of antioxidants and reported to have many bioactivities and health benefits such as antioxidant, anticancer, antimicrobial, anti-inflammatory, hepatoprotective, cardioprotective, neuroprotective, antidiabetic, and anti-obesity activities [44,45]. The main active components in tea are flavonoids such as catechin, epicatechin (EC), epigallocatechin (EGC), epicatechin-3-gallate (ECG), gallocatechin-3-gallate (GCG), epigallocatechin-3-gallate (EGCG), theaflavins, and thearubigins [6,44]. Green tea, oolong tea, and black tea are popularly consumed as beverages in Asian countries [44]. Many studies have demonstrated that traditional kombucha made from black tea or green tea is a potential fermentation product for health promotion. It has been reported that the kombucha fermentation process cause an increase in total polyphenols and flavonoids as well as having antioxidant capacities (Supplementary Table S1) evaluated by using various in vitro antioxidant assays, including 2,2-diphenyl-1-picrylhydrazyl (DPPH), 2,2′-azinobis-(3-Ethylbenzothiazoline-6-sulfonic acid (ABTS), superoxide anion, hydroxyl, and nitric oxide (NO) radical-scavenging activities, ferric-reducing antioxidant power (FRAP), and copper (Cu^2+^)-induced lipid peroxidation inhibitory effects [18,43,46,47,48,49,50,51,52,53,54]. Besides black and green teas, different types of tea obtained from *C. sinensis*, including oolong, red, and white teas, have been used as substrates for the preparation of kombucha [43,52,53]. The results from these studies demonstrated that the chemical profiles, total phenolic content (TPC) and total flavonoid content (TFC), as well as antioxidant abilities, of kombucha depend on the types of tea, fermentation condition, and duration of fermentation.

The antioxidant effects in animal models of traditional kombucha have reported that black tea kombucha could inhibit ROS production and lipid peroxidation, and improve the activities of antioxidant enzymes (e.g., SOD, CAT, GST, GR, and GPx) in alloxan-induced diabetic rats [46]. Treating mice fed a cholesterol-rich diet with traditional black tea kombucha and modified kombucha fermented with *Gluconacetobacter* sp. showed an increase in serum total antioxidant capacity as well as SOD activity and a decrease in malondialdehyde (MDA) [55]. Moreover, the inhibitory effects on lipid peroxidation in chromate (VI)-induced oxidative stress in albino rats was observed by diminishing malondialdehyde (MDA) levels in blood and tissues (liver, lung, kidney, and brain) (Table 1). Meanwhile, the enhancement of GPx and CAT was suppressed by kombucha due to the adaptive response of oxidative stress in chromate-treated rats [56]. Moreover, kombucha could improve immune response by increasing antibody titers and delayed-type hypersensitivity (DTH) response when compared to a control group in rats [56]. In addition, a reduction in thiobarbituric acid-reactive substances (TBARs) and CAT and SOD levels in the livers and kidneys of cholesterol-rich diet-fed rats was also found after oral administration of green tea kombucha (Table 1) [51].

Due to the advantages of kombucha tea over unfermented tea infusions in increasing health benefits and bioactive contents, many medicinal plants, herbs, vegetables, and fruit have been used as substrates for the development of fermented beverages known as kombucha beverages [57]. Many studies revealed the physical, chemical, and biological properties of kombucha beverages made from herbs and medicinal plants (Table 1). Rooibos leaves (*Aspalathus linearis* (Burm. f.) Dahlgren) and yerba-maté leaves (*Ilex paraguariensis* A.St.-Hil.) are popularly used as herbal teas in South Africa and South America, respectively [57]. The TPC and free radical-scavenging activities of the kombuchas made from rooibos tea [54] and yerba-maté tea [58] significantly increased after fermentation for 7 and 12 days, respectively. These herbal tea kombuchas could prevent oxidative cell damage by inhibiting H_2_O_2_-induced oxidative stress in L929 mouse fibroblasts for rooibos kombucha [54] and in *Saccharomyces cerevisiae* BY4741 yeast for yerba-maté kombucha [58]. Another popular beverage worldwide is coffee, which is mostly prepared from arabica (*Coffea arabica* L.) and robusta (*Coffea canephora* Pierre ex A. Froehner) varietals. The main active components in coffee are alkaloids and polyphenols. Moreover, coffee is a rich source of chlorogenic acid, which plays an important role in the antioxidant activity of coffee. One study showed that the preparation of coffee bean kombucha enhanced TPC and antioxidant capacity when tested with the oxygen radical absorbance capacity (ORAC) and DPPH methods during the fermentation process [24]. Meanwhile, another study revealed that fermentation of arabica green coffee for 7, 14, 21, and 28 days resulted in lower TPC, TFC, and DPPH radical-scavenging activity when compared with unfermented green coffee extract. The green coffee kombucha showed the lowest TPC and TFC at 7 days of fermentation. However, the green coffee kombucha at 14 days of fermentation exhibited higher SOD activity than at 7 and 28 days of fermentation and had higher SOD activity than the unfermented extract at a concentration of 0.5 and 1.0 mg/mL [59].

**Table 1:** Antioxidant activities of kombucha beverages made from a variety of raw materials.

Name of Substrates	Active Ingredients	Biological Assays	Findings	Ref.
**In vitro studies**				
Soy milk	▪ Phenolic compounds: gallic acid, catechin, etc. ▪ Isoflavones: daidzin, glycitin ▪ Vitamins: thiamine, riboflavin, etc. ▪ Organic acids: lactic acid, acetic acid, citric acid, etc.	▪ DPPH and ABTS radical-scavenging assay ▪ FRAP assay	▪ DPPH and ABTS radical-scavenging activities as well as FRAP of soy milk kombucha increased more during fermentation at 37 °C than at 28 °C.	[14]
Soy whey	▪ Flavonoids ▪ Isoflavones: glycitin, genistin, glycitein, genistein	▪ DPPH, ABTS, radical-scavenging assay ▪ FRAP assay	▪ Soy whey kombucha showed DPPH and ABTS radical-scavenging activities as well as FRAP (ED_50_ = 1.66, 1.31, 11.34 mg extract/mL) higher than the unfermented sample (ED_50_ = 9.11, 4.58, 821.72 mg extract/mL).	[15]
Coffee	▪ Phenolic compounds: chlorogenic acid, caffeic acid	▪ DPPH radical-scavenging assay ▪ ORAC assay	▪ Coffee kombucha had an increase in antioxidant capacity after fermentation. ▪ ORAC was correlated with TPC more than the other assay.	[24]
Apples, pears, carrots	▪ Phenolic compounds ▪ Organic acids: acetic acid, lactic acid ▪ Vitamins: ascorbic acid, ▪ riboflavin	▪ DPPH, ABTS radical-scavenging assay ▪ FRAP assay ▪ SOD activity assay (xanthine oxidase method using T-SOD assay kit)	▪ DPPH and ABTS radical-scavenging and SOD activities of fermented vegetable-fruit beverage increased to the highest values after fermentation for 8 days.	[48]
Black and green teas and rooibos leaves (*Aspalathus linearis*)	▪ Phenolic compounds ▪ Flavonoids: catechin, epicatechin, gallocatechin, epigallocatechin, etc. ▪ Organic acids: glucuronic acid, acetic acid, etc.	▪ DPPH radical-scavenging assay ▪ FRAP assay ▪ H_2_O_2_-induced oxidative stress in L929 mouse fibroblasts	▪ Antioxidant activity of rooibos kombucha increased at 7th day of fermentation and decreased afterwards. ▪ In curative model, 14-day fermented rooibos kombucha improved cell viability of L929 cells.	[54]
Yerba-maté (*Ilex paraguariensis*)	▪ Phenolic compounds	▪ DPPH and ABTS radical-scavenging assay ▪ H_2_O_2_-induced oxidative stress in *Saccharomyces cerevisiae* BY4741 yeast	▪ DPPH and ABTS radical-scavenging activity was elevated with an increase in fermentation time by 0.5–3.7% and 16.1–31.3% for yerba-maté concentration used. ▪ Yerba-maté kombuchas increased survival rate of *S. cerevisiae* by preventing oxidative cell damage.	[58]
Arabica green coffee	▪ Phenolic compounds: caffeic acid, quinic acid, 3-caffeoylquinic acid, etc. ▪ Alkaloids: caffeine and trigonelline	▪ DPPH radical-scavenging assay ▪ Determination of SOD activity	▪ 7-day- and 28-day-fermented green coffee kombucha had lower DPPH-scavenging activity than unfermented extract. ▪ 14-day-fermented green coffee kombucha had higher SOD activity than 7- and 28-day-fermented kombucha and at concentrations of 0.5 and 1.0 mg/mL had higher SOD activity than the unfermented extract.	[59]
Black tea and Javanese turmeric	▪ Curcuminoids: calebin A, curcumin, demethoxycurcumin, etc. ▪ Conjugated curcuminoids ▪ Terpenoids: xanthorrhizol, bisacurol ▪ Organic acids: acetic acid, carbonic acid, etc. ▪ Vitamin: niacinamide	▪ DPPH radical-scavenging assay	▪ 0.4% Javanese turmeric showed the best properties and highest increase in percentage of antioxidant activity compared to nonfermented extract at the same concentration. ▪ Javanese turmeric kombucha has lower TPC and antioxidant activity than black tea kombucha.	[60]
Black tea and lemon balm	▪ Phenolic compounds of lemon balm kombucha: rosmarinic acid, caffeic acid, quercetin, chlorogenic acid, etc. ▪ Phenolic compounds of black tea kombucha: catechin, gallic acid, epicatechin, etc.	▪ DPPH and hydroxyl radical-scavenging assay	▪ Hydroxyl radical-scavenging activity of kombucha from lemon balm and black tea was higher than unfermented teas. ▪ DPPH-radical-scavenging activity of kombucha from lemon balm and black tea was lower than unfermented teas or tea infusions.	[61]
Black tea and purple basil (*Ocimum basilicum*)	▪ Phenolic compounds ▪ Flavonoids	▪ DPPH and ABTS radical-scavenging assay ▪ CUPRAC assay	▪ Purple basil kombucha had higher inhibitory effects against DPPH (68.09% inhibition) and CUPRAC (43.91% inhibition) than the kombuchas prepared from mixture [3:1], mixture [1:1], and black tea.	[62]
Oolong tea, kitchen mint (*Mentha cordifolia*)	▪ Phenolic compounds	▪ DPPH, ABTS, radical-scavenging assay ▪ H_2_O_2_-induced ROS production in HEK-293 cells ▪ mRNA expression of antioxidant enzymes in HEK-293 cells	▪ All kombuchas exhibited potent DPPH and ABTS radical-scavenging activities. ▪ All kombuchas inhibited H_2_O_2_-induced ROS production in HEK-293 cells. ▪ All kombuchas (0.5% *v*/*v*) caused an increase in mRNA expression of antioxidant enzymes in HEK-293 cells. ▪ All kombuchas increased GRe enzymatic activity in HEK-293 cells.	[63]
Black tea, green tea, winter savory (*Satureja montana*), peppermint (*Mentha×piperita*), stinging nettle (*Urtica dioica*), wild thyme (*Thymus serpyllum*), elderberry (*Sambucus nigra*), and quince (*Cydonia oblonga*)	▪ Organic acids ▪ Phenolic compounds ▪ Flavonoids	▪ Catalase activity ▪ DPPH and hydroxyl radical-scavenging assay ▪ FRAP assay	▪ Winter savory and stinging nettle kombuchas showed the highest catalase activity. ▪ Green tea and wild thyme kombuchas showed the highest DPPH radical-scavenging activity. ▪ Winter savory kombucha showed the highest hydroxyl radical-scavenging activity. ▪ Quince kombucha showed the highest FRAP ability.	[64]
Gum arabic tree *Acacia arabica* (AA), *Aegle marmelos* root bark (AM-RB), *Aerva lanata* (Ala), *Asteracan-tha longifolia* (Alo), *Cassia auriculata* (CA), *Hemidesmus indicus* (HI), *Hordeum vulgare* (HV), *Phyllanthus emblica* (PE), *Tinospora cordifolia* (TC)	▪ Phenolic compounds	▪ DPPH and superoxide radical-scavenging assay ▪ ORAC assay	▪ AM-RB, TC, AA and Ala kombuchas showed significant increase in DPPH radical -scavenging activity on 7th day of fermentation. ▪ ORAC value significantly increased in the kombucha made from AM-RB, TC, AA, Ala, and HV. ▪ While ORAC capacity of the kombuchas ordered from highest to lowest were AM-RB > CA > HI> PE > TC > AA > HV > Alo > Ala.	[65]
Black tea, white oak leaves (*Quercus resinosa*, *Q. arizonica*, *Q. convallata*)	▪ Flavonoids: gallocatechin gallate, gallocatechin, catechin, epicatechin, rutin, quercetin glucuronide ▪ Phenolic acids: gallic acid, 3,4- dihydroxybenzoic acid, etc.	▪ H_2_O_2_ induced-ROS production in macrophages	▪ All concentrations (0.1, 0.2, 2.0 µg/mL) of *Q. resinosa* kombucha showed the same effect in the reduction of oxidative stress. ▪ *Q. arizonica* kombucha inhibited H_2_O_2_ induced-ROS production in a dose-dependent manner (0.1–2.0 µg/mL). ▪ *Q. convallata* and back tea kombuchas showed reduction in oxidative stress at low concentrations (0.1–0.2 µg/mL).	[66]
Oak leaves (*Q. arizonica*, *Q. convallata*)	▪ Hydroxybenzoic acids: gallic acid, etc. ▪ Hydroxycinnamic acids: chlorogenic acid, etc. ▪ Flavan-3-ols: gallocatechin, catechin, etc. ▪ Flavonols: rutin, quercetin 3-O-ß-glucuronide ▪ Dihydrochal: Phloridzin	▪ ABTS radical-scavenging assay ▪ NO scavenging assay ▪ ORAC assay	▪ Oak leaf infusions had more ORAC and ABTS radical-scavenging activity than their kombuchas. ▪ Oak leaf kombuchas exhibited more NO scavenging activity against peroxyl and peroxynitrite anions than their infusions.	[67]
Oolong tea, royal lotus pollen (*Nelumbo nucifera*), butterfly pea flower (*Clitoria ternatea*)	▪ Phenolic compounds	▪ DPPH and ABTS radical-scavenging assay ▪ H_2_O_2_-induced ROS production in HEK-293 cells ▪ mRNA expression of antioxidant enzymes in HEK-293 cells	▪ DPPH and ABTS radical-scavenging activities of all kombuchas significantly increased. ▪ All kombuchas inhibited H_2_O_2_-induced ROS production in HEK-293 cells more than the unfermented teas. ▪ Oolong tea kombucha was less potent than the infusion tea in upregulation of GPx-1, HO-1, and Mn-SOD genes.	[68]
Yarrow (*Achillea millefolium*)	▪ Phenolic compounds ▪ Organic acids: acetic acid, oxalic acid, formic acid, lactic acid, malic acid, succinic acid, citric acid ▪ Vitamin: ascorbic acid	▪ DPPH radical-scavenging assay ▪ FRAP assay	▪ Yarrow flower kombuchas inhibited DPPH with IC_50_ values ranging from 0.02 to 1.12 mg/mL and had reducing power with EC_50_ values ranging from 0.29 to >10 mg/mL. ▪ Kombuchas from subcritical water extracts of yarrow flowers showed antioxidant properties higher than the kombuchas from infusion.	[69]
Black tea and garlic	▪ Phenolic compounds	▪ DPPH radical-scavenging assay	▪ DPPH-scavenging property of black tea kombucha after fermentation with garlic was higher than the fresh kombucha but less than the extracts of kombucha with fermented garlic and fresh garlic.	[70]
Broccoli (*Brassica oleracea*) and spinach (*Amaranthus* spp.)	▪ Phenolic compounds	▪ DPPH radical-scavenging assay	▪ Broccoli and spinach kombuchas showed an increase in antioxidant activity. ▪ Broccoli kombucha had an inhibitory effect against DPPH radicals (75.98% inhibition) higher than spinach kombucha (21.84% inhibition).	[71]
African mustard (*Brassica tournefortii*)	▪ Phenolic compounds	▪ DPPH radical-scavenging assay	▪ EtOAc fraction of African mustard kombucha showed higher DPPH scavenging activity than n-BuOH and aqueous fraction.	[72]
Black carrot (obtained from Konya and Hatay varieties) and green tea	▪ Phenolic compounds ▪ Anthocyanins	▪ DPPH and ABTS radical-scavenging assay ▪ CUPRAC assay	▪ All kombuchas showed an increase in antioxidant capacity tested by DPPH, ABTS, and CUPRAC. ▪ Hatay black carrot kombucha had higher antioxidant capacity than Konya black carrot kombucha and green tea kombucha.	[73]
Black and green teas and laver (*Porphyra dentata*)	▪ Phenolic compounds ▪ Flavonoids	▪ DPPH radical-scavenging assay ▪ FRAP assay	▪ Ultrasound-assisted extraction (UAE)-prepared laver kombucha had higher FRAP than infusion (IE)-prepared laver kombucha but lower than black tea and green tea kombuchas. ▪ Highest DPPH-scavenging activity was observed in UAE-prepared laver kombucha, followed by IE-prepared laver, green tea, and black tea kombuchas.	[74]
Apple varieties (Anna, Fuji, Granny Smith, Manalagi, Red Delicious, Rome Beauty, Royal Gala)	▪ Phenolic compounds	▪ DPPH radical-scavenging assay	▪ Apple kombuchas made from many cultivars had an increase in antioxidant activity during fermentation.	[75]
Snake fruit (*Salacca zalacca*)	▪ Phenolic compounds	▪ DPPH-scavenging assay	▪ Snake fruit kombucha showed an increase in antioxidant activity after the fermentation process.	[76,77]
Cactus pear	▪ Phenolic compounds ▪ Betalains: betacyanin and betaxanthin	▪ DPPH and ABTS radical-scavenging assay	▪ DPPH and ABTS radical-scavenging activities of cactus pear kombucha were significant increased by the 6th day of fermentation by 28.10% and 43.26%.	[78]
Red grape	▪ Phenolic compounds and anthocyanins	▪ DPPH and ABTS radical-scavenging assay	▪ DPPH and ABTS radical-scavenging activities of red grape kombucha were more than nonfermented grape juice by 55.7 and 38.1%.	[79]
Date palm (*Phoenix dactylifera*) fruit and black tea	▪ Phenolic compounds ▪ Organic acids: acetic acid, gluconic acid	▪ DPPH radical-scavenging assay	▪ DPPH radical-scavenging activity of all kombuchas increased during the fermentation process. ▪ Black tea kombucha showed higher DPPH radical-scavenging activity than date syrup kombucha.	[80]
King coconut (*Cocos nucifera* var. aurantiaca)	▪ Phenolic compounds: ferulic acid and *p*-coumaric acid	▪ DPPH and ABTS radical-scavenging assay ▪ ORAC assay ▪ FRAP assay	▪ Antioxidant capacities of coconut water kombucha significantly increased during the fermentation process.	[81]
Red goji berry (*Lycium barbarum*), black goji berry (*Lycium ruthenicum*), and black tea	▪ Phenolic compounds	▪ DPPH radical-scavenging assay ▪ CUPRAC assay ▪ FRAP assay	▪ CUPRAC of black goji berry kombucha was higher than red goji berry kombucha but lower than black tea kombucha. ▪ There was no significant difference in DPPH-scavenging activity between kombuchas. ▪ FRAP values of the kombuchas made form black tea, black goji berry, and red goji berry increased during fermentation.	[82]
Acerola	▪ Phenolic compounds ▪ Vitamin C	▪ DPPH radical-scavenging assay	▪ Kombuchas made from 3 and 5% acerola byproduct showed significant increase in DPPH radical-scavenging effect by 14.8% and 6.6%, respectively, which related to an increase in vitamin C content.	[83]
Green tea, pitanga (*Eugenia uniflora*), and umbu-caja’ (*Spondia tuberosa*) fruit	▪ Phenolic compounds: syringic acid, caftaric acid, chlorogenic acid, caffeic acid, procyanidin B2, myricetin, etc. ▪ Organic acids: acetic acid, citric acid, malic acid, buryric acid, etc.	▪ DPPH radical-scavenging assay ▪ ORAC assay	▪ Pitanga and umbu-caj’ pulp-flavored kombuchas had higher antioxidant activities than green tea kombucha at 0 days of storage. ▪ After storage for 7 days, the highest DPPH-scavenging activity and ORAC were observed in green tea kombucha and pitanga-flavored kombucha.	[84]
**In vivo studies**				
Black tea	▪ Phenolic compounds	▪ Studies in pancreatic, hepatic, renal and cardiac tissues of alloxan-induced diabetic rats ▪ Tissue ROS level determined by using DCFH-DA ▪ Determination of lipid peroxidation by measurement of TBARS ▪ Assay of synthesis of antioxidant enzymes	▪ Kombucha showed more effective in the suppression of ROS, TBARS, and protein carbonyls formation in the tissues. ▪ Kombucha improved the activities of antioxidation enzymes in the tissues of alloxan-induced diabetic rats with higher effects than nonfermented tea by increasing CAT and SOD in pancreas and kidneys, decreasing CAT and SOD in heart and liver, and increasing GST, GRe, and GPx in all tissues.	[46]
Green tea	▪ Phenolic compounds	▪ ABTS assay (in serum of cholesterol-rich diet-fed rats) ▪ TBARS measurement (MAD levels in liver and kidneys of cholesterol-rich diet-fed rats) ▪ Antioxidant enzyme activities (CAT, and SOD in liver and kidneys of cholesterol-rich diet-fed rats)	▪ Kombucha and unfermented tea reduced TBARS, CAT, and SOD levels in liver and kidneys of cholesterol-rich diet-fed rats. ▪ TBARS levels in liver and kidneys decreased by 55 and 44% in kombucha and cholesterol-rich diet-fed rats when compared with rats fed only cholesterol-rich diet. ▪ Treatment of cholesterol-rich diet-fed rats with kombucha and unfermented tea attenuated CAT and SOD activities in liver and kidneys.	[51]
Black tea	▪ Phenolic compounds	▪ Antioxidant enzymatic activity (TOAC, SOD, and MDA level in serum of cholesterol-rich diet-fed mice)	▪ In serum of cholesterol-rich diet-fed mice, TOAC and SOD activities were decreased by 45.4 and 52.6%, respectively, and MDA increased by 189.8%. ▪ Treatment cholesterol-rich diet-fed mice with traditional and modified kombuchas (fermented with *Gluconacetobacter* sp.) increased TOAC and SOD activities, as well as a decreased MDA in serum.	[55]
Black tea	▪ Polyphenols and flavonoids	▪ Chromate (VI)-induced oxidative stress in rats ▪ Determination of MDA in blood and tissues ▪ Determination of GSH and antioxidant enzymes in blood	▪ Kombucha reduced MDA in plasma and tissues of chromate treatment. ▪ Kombucha reversed the physiological effects of chromate by suppressing the enhancement of GPx and CAT.	[56]
Oak leaves (*Q. arizonica* and *Q. convallata*)	▪ Hydroxybenzoic acids: gallic acid, salicylic acid, etc. ▪ Hydroxycinnamic acids: chlorogenic acid, etc. ▪ Flavan-3-ols: gallocatechin, catechin, gallocatechin gallate ▪ Flavonols: rutin, quercetin 3-O-ß-glucuronide ▪ Dihydrochal: phloridzin dihydrate	▪ ABTS radical-scavenging assay ▪ NO scavenging assay ▪ ORAC assay	▪ Oak leaf kombuchas did not show any differences in ABTS and ORAC activity in blood samples of high saturated fat and fructose diet-induced obesity mice. ▪ Oak leaf infusions and kombucha allowed for *Q. convallata* kombucha improved antioxidant capacity when testing the blood of obesity mice by NO scavenging assay.	[67]
Snake fruit (*S. zalacca*) and black tea	▪ Phenolic compounds	▪ SOD activity and MDA levels in streptozotocin-induced diabetic rats	▪ Snake fruit and black tea kombuchas improved SOD activity and diminished MDA levels in diabetic rats	[77]

A variety of medicinal plants, herbs, and spices, including Javanese turmeric rhizomes (*Curcuma xanthorrhiza* Roxb.) [60], winter savory herbs (*Satureja montana* L.), lemon balm leaves (*Melissa officinalis* L.) [61], purple basil (*Ocimum basilicum* L.) [62], kitchen mint (*Mentha cordifolia* Opiz. Ex Fresen) [63], wild thyme (*Thymus serpyllum* L.), peppermint leaves (*Mentha × piperita* L.), stinging nettle leaves (*Urtica dioica* L.), quince leaves (*Cydonia oblonga* Mill.), elderberry flowers (*Sambucus nigra* L.) [64], gum arabic tree (*Vachellia nilotica* subsp. *tomentosa* (Benth.) or *Acacia arabica* (Lam.) Willd., bael root bark (*Aegle marmelos* (L.) Corrêa), mountain knotgrass (*Ouret lanata* (L.) Kuntze or *Aerva lanata* (L.) Juss. ex Schult.), marsh barbel (*Hygrophila auriculata* (Schumach.) Heine or *Asteracantha longifolia* (L.) Nees.), avaram senna (*Senna auriculata* (L.) Roxb. or *Cassia auriculata* L.), Indian sarsaparilla (*Hemidesmus indicus* (L.) R.Br.), cebada (*Hordeum vulgare* L.), Indian gooseberry (*Phyllanthus emblica* L.), heart-leaved moonseed (*Tinospora cordifolia* (Willd.) Hook.f. & Thomson) [65], oak leaves (*Quercus resinosa* Liebm., *Q. convallata* Trel., and *Q. arizonica* Sarg.) [66,67], royal lotus pollen (*Nelumbo nucifera* Gaertn.), butterfly pea flowers (*Clitoria ternatea* L.) [68], roselle (*Hibiscus sabdariffa* L.) [85], and yarrow flowers (*Achillea millefolium* L.) [69] have been used as substrates for the preparation of kombuchas. Some of the alternative kombucha beverages have been reported to increase TPC and TFC during fermentation process [63,65,68]. The results are related to the effect of fermentation on the chemical components of the traditional kombucha.

Most kombuchas derived from herbs and medicinal plants exhibited free radical-scavenging properties against DPPH, ABTS, hydroxy, and superoxide radicals as well as ORAC which were enhanced during the fermentation process. Moreover, the kombuchas made from kitchen mint leaves, oolong tea leaves, butterfly pea flowers, and mixtures of oolong tea and kitchen mint were found to possess an intracellular antioxidant effect by protecting against H_2_O_2_-induced ROS production in human embryonic kidney 293 (HEK-293) cells [63,68]. The oolong tea and butterfly pea kombuchas could induce the synthesis of antioxidant enzymes, including CAT, GPx-1, GR, manganese superoxide dismutase (Mn-SOD), and heme oxygenase-1 (HO-1) in HEK-293 cells via the upregulation of their mRNA expression [68]. Oak leaf (*Q. arizonica*) kombucha has been reported to have an oxidative stress inhibitory effect by attenuating H_2_O_2_-induced ROS production in macrophages in a dose-dependent manner [66]. Moreover, feeding fermented beverages from *Q. convallata* (200 μL/per day) for 3 months could reduce oxidative stress in high-saturated fat and -fructose diet-induced obesity in female C57BL/6 mice by diminishing nitric oxide (NO) in the blood of the obese mice [67].

It is interesting that after fermentation of garlic bulbs (*Allium sativum* L.) with black tea kombucha or grape vinegar, the TPC and antioxidant activity of the fermented garlic bulbs was lower than fresh garlic extract. The garlic fermented with kombucha had higher TPC and antioxidant activity than garlic fermented with grape vinegar. Meanwhile, the black tea kombucha after fermentation with garlic had higher TPC and DPPH-scavenging activity than fresh kombucha but lower than kombucha-fermented garlic and fresh garlic extracts, respectively [86]. Other vegetables such as broccoli (*Brassica oleracea* L.), spinach (*Amaranthus* spp.) [71], African mustard (*Brassica tournefortii* Gouan or *Coincya tournefortii* (Gouan) [72], carrot and black carrot (*Daucus carota* subsp. sativus) [48,73], and laver (*Porphyra dentata* Kjellman), a red seaweed [74], were used as substrates for fermentation of kombuchas. These studies also reported an increase in free radical-scavenging activity and reduced antioxidant capacity, which related to the elevation of the active compounds in each plant, such as polyphenols, flavonoids, and anthocyanins, in the kombucha-analogues during the fermentation process as shown in Table 1.

Various fruit kombuchas have been developed and investigated for their antioxidant effects. Apple (*Malus domestica* (Suckow) Borkh.) [48,75], pear (*Pyrus* spp.) [48], snake fruit (*Salacca zalacca* (Gaerth) [76,77], cactus pear (*Opuntia ficus-indica* (L.) Mill.) [78], red grape (*Vitis vinifera* L.) [79], date palm (*Phoenix dactylifera* L.) [80], king coconut water (*Cocos nucifera* var. aurantiaca) [81], red goji berry (*Lycium barbarum* L.), black goji berry (*Lycium ruthenicum* Murr.) [82], and acerola (*Malpighia emarginata* DC.) byproducts from the juice clarification step [83] were reported as alternative substrates of kombuchas as shown in Table 1. The fermentation process is also beneficial for the enhancement of antioxidant contents such as polyphenols, flavonoids, and tannins, together with the free radical-scavenging effect and antioxidant-reducing power of the fruit kombuchas. Moreover, the effects of snake fruit and black tea kombuchas on the reduction of oxidative stress in diabetic rats have been studied. The administration of 5 mL/kg bw/day of the kombuchas or 45 mg/kg bw/day of metformin to streptozotocin-induced diabetic rats for 28 days resulted in an increase in SOD activities and a decrease in MDA levels when compared with untreated diabetic rats [77]. In addition, one study showed that favoring a green tea kombucha with 15% pulp from pitanga (*Eugenia uniflora* L.), or umbu-caja’ (*Spondia tuberosa*) fruit caused an increase in antioxidant activity. However, favored kombuchas had a slight decrease in DPPH-scavenging activity and an increase in ORAC after storage for 7 days [84].

Soymilk [14], a soybean (*Glycine max* (L.) Merr.) product, and soy whey [15], a liquid byproduct from soybean processing, were developed as fermented health beverages using kombucha consortium (Table 1). These studies demonstrated that soy kombucha beverages had higher phenolic and flavonoid contents than their unfermented counterparts. Furthermore, chemical analysis showed that the contents of isoflavone glycosides (e.g., daidzin, glycitin, and genistin) decreased while the content of isoflavone aglycones (e.g., daidzein, glycitein, and genistein) increased after microbial fermentation. The obtained results were due to the transformation of flavonoid glycosides into flavonoid aglycones by β-glucosidase secreted from the organisms during the fermentation process. The antioxidant capacity assessed by DPPH, ABTS radical-scavenging, and FRAP assays of kombucha-fermented soymilk or soy whey significantly increased relative to their active contents [14,15].

According to many studies that have been mentioned, the increase in TPC and TFC in kombuchas prepared from different substrates during the fermentation were associated with antioxidant and other biological activities in the kombucha beverages. Yeasts and bacteria contained in kombucha cultures are important for bioconversion of the chemical constituents in substrates. During microbial fermentation, many biochemical reactions occur and are catalyzed by some microorganism enzymes which cause the degradation of polyphenol complexes or conjugated forms into small molecules of phenolic compounds such as phenolic acids and flavonoid aglycones [43,46,49]. Apart from enzymatic action, acidic fermentation conditions can facilitate the degradation and hydrolysis of complex molecules [15,49]. The chemical degradation caused by the fermentation process, as well as the ability of the microorganisms to liberate bioactive compounds from plant matrices and other components, causes the elevation of the contents of phenolic and other active compounds [46,60]. Consequently, kombucha allows an increase in bioavailability and biological activities, especially for antioxidant properties leading to improved benefits of health beverages [60].

### 3.2. Anti-Inflammatory and Immunomodulatory Activities

When cells and tissues are injured by pathogens, irritants, toxic chemicals, or any other causes, inflammation is the immune system’s first-line response in removing the harmful stimuli and promoting the healing process [7,87]. The inflammation response involves the activation of signaling pathways which regulate various inflammatory cells and mediators and other biological molecules [7]. Acute inflammation is a cause of redness, swelling, heat, pain, and impairment of tissue function after injury or infection within minutes to hours due to the activation of leukocyte chemotaxis, inflammatory mediator production and release, and changes in vascular permeability [7,87]. Normally, the effects from acute inflammatory response can be healed within a few days. If the inflammation response is prolonged and continuously activates the immune system, various chronic diseases such as rheumatoid arthritis, inflammatory bowel disease, multiple sclerosis, asthma, allergy, diabetes, cardiovascular disease, neurodegenerative disease, and cancer will develop [7,87,88,89,90].

In the inflammation process, the inflammatory cells such as leukocytes, endothelial cells, and macrophages are activated to produce proinflammatory cytokines, including interleukin (IL)-1*β*, IL-6, IL-17, and tumor necrosis factor-alpha (TNF-α), as well as anti-inflammatory cytokines such as IL-4, IL-10, and IL-13 [87,91]. Moreover, they also produce proinflammatory enzymes, such as cyclooxygenase (COX) and inducible nitric oxide synthase (iNOS), which are essential for the synthesis of proinflammatory mediators, prostaglandins (PEG_2_), and nitric oxide (NO), respectively. An increase in the levels of the proinflammatory mediators and enzymes is associated with acute and chronic inflammation [87,91].

Vegetables, fruit, whole grains, beans, nuts, herbs, spices, and mushrooms are important sources of anti-inflammatory and immunomodulatory components such as polyphenols, terpenoids, and polysaccharides [90,92,93,94,95]. It is well known that kombucha beverages have been developed from a variety of plants and contain a great deal of polyphenols, including gallic acid, catechins, epicatechins, and flavonoids [6,47,56,96,97], which may help to ameliorate inflammatory reactions and immune function by modulating inflammatory molecules and oxidative stress [90,92,94]. The traditional kombuchas prepared from black tea and green tea were evaluated for anti-inflammatory activities via in vitro and in vivo experiments. The kombucha was found to have higher inhibitory activity against the enzyme 5-lipoxygenase than unfermented tea infusion did [47] (Table 2). Lipoxygenases (LOXs) are oxidative enzymes in the metabolism of polyunsaturated fatty acids, such as arachidonic acid. Arachidonic acid metabolites such as leukotrienes are proinflammatory mediators related to many physiological processes, including inflammation, cardiovascular disease, and cancer progression [97,98]. Moreover, kombuchas from black tea and green tea exhibited anti-inflammatory effects by inhibiting the production of many proinflammatory mediators, including IL-1α, IL-6, TNF-α, and NO, in lipopolysaccharide (LPS)-induced inflammatory responses in RAW264.7 macrophages [96].

Many studies investigated the anti-inflammatory and related activities of kombucha beverages in animal models as shown in Table 2. Black tea kombucha exhibited ulcer-healing by protecting mucin content and decreasing gastric acid secretion. Black tea kombucha at a dose of 15 mg/kg had an ulcer-healing effect the same as omeprazole, an anti-ulcer drug, at a dose of 3 mg/kg when treating indomethacin-induced gastric ulcers in mice. The increase in the content of phenolic compounds and organic acids in the kombucha may be beneficial for healing due to the suppression of oxidative stress, inflammation, and tissue damage [99]. Another study showed that oral administration of green tea kombucha could increase the survival rate and thermoregulation of LPS-induced sepsis in mice [97]. Moreover, the anti-inflammatory and immunomodulatory effects of green tea kombucha were associated with a reduction in TNF-α, IL-1β, and IL-6 levels, the recovery of T-cells, and macrophage levels in LPS-challenged mice, as well as the alleviation of tissue damage and the inhibition of NF-κB signaling in LPS-induced sepsis in mice [97]. Improvement in gut microbiota diversity and butyrate-producing bacterial growth were found to be other promoting factors for anti-inflammatory effects of the kombucha tea [97].

The efficacy of black tea kombucha on multiple sclerosis (MS), an inflammatory disorder of the central nervous system, has been tested and was found to attenuate the incidence and severity, as well as delay onset of the disease in experimental autoimmune encephalomyelitis (EAE) mice [100]. A decrease in inflammatory criteria such as infiltrated immune cells and plaque levels, demyelination, and serum levels of NO and TNF-α were observed in kombucha tea-treated mice more than in the control group [100]. Another study showed that kombucha significantly reduced EAE clinical symptoms and the productions of IL-17, interferon-γ and NO and elevated the production of the anti-inflammatory mediators IL-4 and TGF-β in oligodendrocyte glycoprotein (MOG_35–55_)-induced EAE mice. Therefore, kombucha tea may be beneficial for the treatment of MS [89].

The in vivo immunomodulatory activity of the traditional kombucha and alternative kombuchas from arabica coffee [101] and turmeric (*Curcuma longa* L.) [102] was evaluated in *Salmonella typhi*-infected Balb-C mice. The results showed that administration of the kombuchas enhanced the adaptive immune response and innate immune response. The bioactive components such as flavonoids, tannins [101], and curcuminoids [102] found in the plans act as immunomodulatory agents which play an important role in the immunomodulatory activity of kombuchas (Table 2). The anti-inflammatory and immune modulatory activities of alternative kombuchas from oak leaves (*Q. resinosa*, *Q. arizonica*, and *Q. convallata*) have been shown to have the ability to suppress TNF-α and IL-6 production as well as NO in macrophages stimulated by LPS [66] (Table 2).

The medicinal mushrooms turkey tail (*Coriolus versicolor* (L.) Quél.) [103] and shiitake (*Lentinus edodes* (Berk.) Pegler) [104] are interesting as nutraceuticals due to their biological activities, such as antioxidant, anticancer, and immunomodulatory activities. The fruiting bodies of the mushrooms were used as substrates for preparing fermented health beverages. It was found that *C. versicolor* kombucha extract (CVex) had more polysaccharides, phenols, and flavonoids than *L. edodes* kombucha extract (LEex). The polysaccharide extracts of mushroom kombuchas were tested for immunological properties in phytohemagglutinin-activated peripheral blood mononuclear cells. The LEex induced the synthesis of some proinflammatory cytokines, TNF-α, IL-6 and IL-8, while the CVex increased TNF-α and IL-8 production. The strongest effect of both extracts was observed in the suppression of IL-4 and IL-5 synthesis. Both mushroom kombuchas exhibited antiallergic activity via the inhibition of the production of IL-4, IL-5, and IL-10, which are associated with allergic reactions [95] (Table 2). Based on the results of the previous studies, drinking kombucha beverages might be beneficial for our health and support the immune system. Due to their potential anti-inflammatory and immunomodulatory effects, kombucha beverages could prevent and attenuate the severity of allergies, infections, and some inflammatory disorders.

### 3.3. Antimicrobial Activities

Infectious diseases are significant causes of disability and mortality worldwide. Various pathogens, including bacteria, fungi, and viruses, cause human illnesses by many routes such as inhalation, skin contact, and consumption of contaminated food or water. The attempt to investigate for new anti-infective agents has been reported due to an increase in antimicrobial resistance [16,88]. Natural antimicrobial agents have been found in plants, microorganisms, and animals. Plants, especially herbs, spices, fruit, and vegetables, are rich sources of phenolic compounds, essential oils, and organic acids which possess antimicrobial properties against human pathogens [105]. Kombuchas contain high contents of phenolic compounds and organic acids which increase during the fermentation process [7,15,75,76,88,106,107,108]. Many studies have revealed antimicrobial properties against bacteria and fungi in kombuchas prepared from tea, together with a variety of raw materials (Table 3).

Traditional kombuchas made from black tea and green tea have been reported to exert antimicrobial properties against pathogenic bacteria and fungi. Black tea and green tea kombucha could inhibit the growth of both Gram-positive (e.g., *Staphylococcus aureus*, *Staphylococcus epidermis*, *Bacillus cereus*, *Listeria monocytogenes*, and *Micrococcus luteus*) and Gram-negative bacteria (e.g., *Escherichia coli*, *Pseudomonas aeruginosa*, *Enterobacter cloacae*, *Shigella sonnei*, *Shigella dysenteriae*, *Salmonella enteritidis*, *Salmonella enterica*, *Salmonella typhimurium*, *Salmonella typhi*, *Aeromonas hydrophila*, *Yersinia enterolitica*, *Campylobacter jejuni*, *Haemophilus influenzae*, *Helicobacter pylori*, and *Vibrio cholerae*) as shown in Table 3.

Antibacterial activity in oolong tea kombucha has been reported against *E. coli*, *S. dysenteriae*, *S. typhi*, and *V. cholerae* [52,110]. Moreover, the addition of cinnamon, cardamom, or Shirazi thyme extracts in green tea kombucha caused a change in antibacterial activity against *S. aureus*, *B. cereus*, *E. coli*, and *S. typhimurium*. Among those, cinnamon-flavored kombucha exhibited the highest antibacterial activity against *E. coli* and *S. typhimurium* and the activity was associated with the concentration of cinnamon extract in the kombucha [111] (Table 3). Apart from antibacterial activity, black tea and green tea kombuchas have been shown to possess antifungal properties against candida yeasts (*Candida albicans*, *Candida krusei*, *Candida tropicalis*, *Candida parapsilosis*, *Candida glabrata*, *Candida dubliniensis*, and *Candida sake*) [107,109,115] as well as the other pathogenic fungi such as *Aspergillus flavus*, *Aspergillus niger*, and *Microsporum gypseum* [106,112] (Table 4).

A variety of fruit kombuchas have been investigated for their antimicrobial activities as shown in Table 3. Mulberry kombucha showed antibacterial effects against *V. cholerae* [110]. Cactus pear and red grape kombuchas exerted antibacterial activity against both Gram-positive bacteria (*S. aureus*, *S. epidermidis*, *B. cereus*, and *Enterococcus faecalis*) and Gram-negative bacteria (*E. coli*, *P. aeruginosa*, and *Klebsiella pneumoniae* [78,79]. Snake fruit and apple kombuchas were reported to have better antibacterial properties against *S. aureus* and *E. coli* than fruit juices [75,76]. Moreover, antimicrobial activity has been observed in kombuchas prepared from other substrates as shown in Table 3. The kombucha made from yarrow flowers could inhibit the growth of bacteria and fungi, including *S. aureus*, *Bacillus subtilis*, *E. coli*, *K. pneumoniae*, *P. vulgaris*, *P. mirabilis*, *C. albicans*, and *A. niger* with minimum inhibitory concentration (MIC) values (from 9.77 to 312.50 µg/mL) [69].

The kombucha produced from lemon balm leaves showed antimicrobial activity against *S. aureus*, *S. enteritidis*, *B. cereus*, *P. aeruginosa*, *P. mirabilis*, *E. coli*, *Citrobacter freundii*, and *Erwinia carotovora* but did not affect any tested fungi [61,112]. Antimicrobial activity of kombuchas made from herbs, including thyme (*Thymus vulgaris* L.), lemon verbena (*citriodora* Kunth), rosemary (*Rosmarinus officinalis* L.), fennel (*Foeniculum vulgare* Mill.), and peppermint has been reported. All fermented herb extracts apart from thyme had antibacterial properties against *S. aureus*, *S. epidermidis*, *L. monocytogenes*, *M. luteus*, *E. coli*, *P. aeruginosa*, and *S. typhimurium* [108,113] (Table 3). In addition, lemon verbena and peppermint kombuchas exhibited antimicrobial properties against *B. cereus*, and *S. dysenteriae* [108], whereas anti-candida activity against *C. glabrata*, *C. tropicalis*, *C. sake*, *C. dubliniensis*, and *C. albicans* was observed only in the kombuchas made from lemon verbena, fennel, and peppermint [113]. Antibacterial activity was also found in the kombuchas made from beet (*Beta vulgaris* L.) (against *E. coli*) [114], yerba-maté (against *S. aureus* and *E. coli*) [58], and soy whey (against *S. aureus*, *B. subtilis* and *E. coli*) [15]. However, one study reported that antibacterial activity against *S. saprophyticus*, *S. aureus*, *S. epidermidis*, *B. stearothermophilus*, *S. typhimurium*, *E. coli*, and *P. aeruginosa* was not observed in fresh black tea kombucha and kombucha after fermentation with garlic, whereas garlic fermented in kombucha exhibited antibacterial activity against all tested bacteria but was less active than fresh garlic extract, which had stronger inhibitory effect against *S. aureus* than the standard antibiotics gentamycin and amoxicillin [70].

**Table 4:** Antifungal and antiviral activities of kombucha tea and kombucha beverages made from a variety of raw materials.

Name of Substrates	Active Ingredients	Biological Assays	Findings	Ref.
**In vitro studies of antifungal activities**				
Black and green teas	▪ Organic acids	▪ Agar disc diffusion (fermented infusion, unfermented infusion, neutralized kombucha)	▪ All samples had no activity against *C. albicans.*	[23]
Yarrow (*Achillea millefolium*)	▪ Phenolic compounds ▪ Organic acids: xalic acid, formic acid, acetic acid, lactic acid, succinic acid, malic acid, citric acid, etc.	▪ Microdilution method in 96-multi-well microtiter plates	▪ Kombuchas showed antifungal activity against *C. albicans* and *A. niger* (MIC = 39.10 − 312.50 µg/mL)	[69]
Black tea	▪ Heterocyclic compounds ▪ Lactones ▪ Antibiotics ▪ Unsaturated fatty acid ▪ Alkaloids: caffeine	▪ Agar well diffusion (fermented infusion, unfermented infusion, neutralized kombucha, heat-denatured kombucha)	▪ Kombucha showed antifungal activity against *A. flavus* and *A. niger* higher than heat-denatured kombucha and neutralized kombucha. ▪ Unfermented tea had no antifungal activity.	[106]
Black and green teas	▪ Organic acids	▪ Agar well diffusion (fermented infusion, unfermented infusion, acidified infusion, neutralized kombucha, heat-denatured kombucha)	▪ Kombuchas showed anti-Candida activity against *C. glabrata*, *C. tropicalis*, *C. sake*, *C. dubliniensis*, and *C. albicans.* ▪ Acidity is important for antifungal activity. ▪ All neutralized kombuchas and some heat-denatured kombuchas had lower antifungal activity than untreated kombucha.	[107]
Black tea	▪ Phenolic compounds ▪ Organic acids: acetic acid, tartaric acid, etc.	▪ Agar disc diffusion (kombucha, unfermented sweet tea)	▪ Kombucha showed potent antifungal activity against *C. krusei*, *C. glabrata*, *C. albicans*, and *C. tropicalis.*	[109]
Lemon balm (*Melissa officinalis*)	▪ Organic acids: acetic acid	▪ Agar well diffusion (fermented infusion, unfermented infusion, acidified infusion, neutralized kombucha, heat-denatured kombucha)	▪ All tested samples did not have antifungal activity against *C. pseudotropicalis*, *Rhodotorula sp*., *A. flavus*, *A. niger*, or *P. aurantiogriseum.*	[112]
Thyme (*Thymus vulgaris*), lemon verbena (*Lippia citriodora*), rosemary (*Rosmarinus officinalis*), fennel (*Foeniculum vulgare*), and peppermint (*Mentha piperita*)	▪ Organic acids	▪ Agar well diffusion (fermented infusion, unfermented infusion, acidified infusion, neutralized kombucha, heat-denatured kombucha)	▪ All kombuchas (except for rosemary and thyme kombucha) showed anti-candida activity against *C. glabrata*, *C. tropicalis*, *C. sake*, *C. dubliniensis*, and *C. albicans.* ▪ All neutralized kombuchas had lower antifungal activity than untreated kombucha.	[113]
Black tea	▪ Organic acids: acetic acid	▪ Agar disc diffusion (kombuchas fermented for 6, 12, and 18 days)	▪ All kombuchas showed antifungal activity against *A. flavus*, *C. albicans*, and *M. gypseum*.	[115]
**In vivo studies of antiviral activities**				
Licorice (*Glycyrrhizae Radix*), Grosvenor Momordica (*Momordica Grosvenori*), Chry-santhemum (*Dendranthema morifolium*), and green tea (*CamelliaSinensis*)	▪ Not determined	▪ Foot and mouth disease (FMD) challenge in swine, daily oral administration and spraying mouth and nose with different doses of Chinese herbal kombucha (1:1, 1:3, 1:11 dilution) for 13 days, starting three days before the viral challenge ▪ Field trial: small outbreak of FMD in cattles, daily spraying of mouth and nose with 1:4 dilution of the kombucha for six days	▪ Chinese herbal kombucha at low dose (1:11 dilution) prevented FMD typical clinical symptoms, inhibited FMDV replication, and no viral genome copies were detected. ▪ Field trial: kombucha-treated animals did not show FMD symptoms after treatment for two weeks.	[116]

Besides antibacterial and antifungal activities, it was reported that antiviral activity of kombucha was investigated in a foot-and-mouth disease (FMD) challenge study of swine as shown in Table 4. Oral administration and spraying the mouth and nose with Chinese herbal kombucha made from a mixture of green tea, licorice, Grosvenor Momordica (*Momordica grosvenori* Swingle), and chrysanthemum (*Dendranthema × morifolium* (Ramat.) Tzvelev) resulted in the inhibition of FMD symptoms and the virus. Moreover, the kombucha could prevent FMD symptoms after spraying mouth and nose of cattle during a small FMD outbreak [116].

According to the literature on antimicrobial activity of kombucha beverages, many studies attempted to obtain more information about the important factors that influence antimicrobial properties. A number of studies compared the antimicrobial activity of different types of kombucha preparations, including fermented infusion, unfermented infusion, acidified infusion, neutralized kombucha, and heat-denatured kombucha as shown in Table 3 and Table 4. The results of most studies demonstrated that the fermented infusion of kombucha and acidified infusion exerted antimicrobial activity against various pathogenic bacteria and fungi more than neutralized kombucha and unfermented counterparts. This finding indicated that organic acids, especially acetic acid, are considered to be an influential factor for antimicrobial activity. The role of acetic acid in antimicrobial activity is associated with the ability to diminish pH value via proton releasing. This effect leads to the inhibition of pathogenic microbial growth by changing cell membrane permeability and disturbing cell membrane function and enzymatic activity [7,16,75,106,108].

Some studies have reported the inhibitory effect against pathogenic microorganisms of neutralized kombucha while it was not observed in most of the unfermented samples. These results suggested that apart from organic acids, other bioactive compounds in kombucha such as flavonoids, tannins, proteins, and bacteriocins generated during fermentation may be responsible for antimicrobial effects [7,15,88,106,107,108]. In addition, the antimicrobial activity of heat-denatured kombucha showed no significant difference or a slight decrease when compared with the kombucha before heating. Therefore, antimicrobial components are stable at high temperature [61,106,107,117].

Furthermore, studies on the efficiency of kombucha prepared with different fermentation times showed that antimicrobial activity increased with time of fermentation due to the increase in the production of acetic acid, other organic acids, and other metabolites, as well as the low pH value or acidic property during the fermentation process [15,75,79,96,108,110,113,115]. Besides the fermentation time, sugar concentration and type of fermented substrates are important factors for increasing antimicrobial activity as well as the content of bioactive components, especially organic acids and phenolic compounds (Table 3). Sugar is necessary for fermentation. It is changed into ethanol, which is further converted into acetic acid and other organic acids by yeasts and acetic acid bacteria in the kombucha culture, respectively. Therefore, increasing sugar concentration results in the elevation of organic acid content in kombucha beverages [108].

Moreover, the presence of other antimicrobial metabolites is dependent on the type of substrate and kombucha culture. Consequently, many factors need to be considered for the development of the fermented health beverages in order to improve their biological activities. Due to a variety of health benefits of kombucha, including antimicrobial activity, the fermented beverages are considered alternative sources of many bioactive compounds for preventing some infectious diseases and promoting health [16,106].

### 3.4. Anticancer Activities

Cancer is the second leading cause of death worldwide. According to a recent study by Ferlay and coworkers, there were an estimated 10 million cancer deaths and 19.3 million new cases of cancer in 2020 [117]. Cancer is characterized by the uncontrolled growth and spread of abnormal cells, which can invade surrounding tissue and eventually metastasize to other parts of the body, resulting in undesired symptoms and even death. Some cancer treatments at present still lack specificity and cause side effects. Therefore, there is a constant effort to develop more effective therapeutic strategies to overcome treatment limitations and improve therapeutic outcomes.

Numerous studies have demonstrated the anticancer properties of kombucha through a variety of mechanisms, with antiproliferative activity being extensively studied and proven in several cancer cell lines as shown in Table 5. The cytotoxicity of kombucha, as observed in in vitro experiments, has been found to vary depending on the type of cancer cells but has a more pronounced effect in cancer cells compared to normal cells [49,52]. For instance, kombucha prepared from green and black teas has been found to possess potent cytotoxicity against colorectal cancer Caco-2 cells, as evidenced by its lower IC_50_ value compared to that of normal NIH/3T3 fibroblasts, as kombucha contains various organic acids which have been identified as potential anticancer substances such as acetic acid, gluconic acid, glucuronic acid, lactic acid, and ascorbic acid [52].

Kombucha fractionated with different solvents including chloroform, ethyl acetate, and butanol have been found to exhibit varying levels of inhibition on the invasiveness and motility of cancer cells. In particular, kombucha fractioned with ethyl acetate showed the strongest inhibitory effect on cell invasion and cell motility in human lung carcinoma (A549), human osteosarcoma (U2OS), and human renal carcinoma (786-O) cells, as well as a reduction in the activity of metalloproteinases (MMPs), including MMP-2 and MMP-9, which are the molecules that are important for cancer invasion and migration, in 786-O cells [118] (Table 5). In this case, dimethyl 2-(2-hydroxy-2-methoxypropylidene)malonate and vitexin that were purified from ethyl acetate of kombucha are suggested as being responsible for these inhibitory effects [118]. Furthermore, treatment with kombucha (lyophilized) downregulated the expression of angiogenic stimulating genes including HIF-1α, VEGF, IL-8, and COX-2, which play a role in the formation of new blood vessels at the tumor site, as well as reduced expression of MMP-2 and MMP-9 in prostate cancer (PC-3) cells in a dose-dependent manner. Therefore, kombucha may prevent prostate cancer cell survival as it inhibited angiogenesis and metastasis potential which are the crucial steps in cancer progression [119].

The anticancer properties and chemical composition of kombucha are varied depending on the types of kombucha substrates used and fermentation conditions (Table 5). Different types of kombucha, such as green and black tea, have different effects on the growth inhibition of lung cell carcinoma (A549) and epidermoid carcinoma (Hep-2) cells [120]. Interestingly, a previous study found that green tea kombucha had greater inhibitory effects on cancer cell proliferation than black tea kombucha, which may be related to the fact that green tea contains high concentrations of catechins and verbascoside [49]. Using two distinct vessel geometries with low or high surface/height ratios for kombucha preparation produced different fermentation metabolites and bioactive compounds, leading to varying levels of inhibition in human breast cancer (MCF-7) and human colon cancer (HCT-116) cells. Notably, the highest inhibition was observed in HCT-116 cells treated with kombucha fractioned with ethyl acetate and prepared in vessels with high surface-to-height ratios [47].

The yarrow kombucha obtained from two substrate preparation techniques, including infusion and subcritical water extraction, resulted in varying levels of total phenols, flavonoids, and organic acids [69]. These differences in chemical composition led to differing levels of antiproliferative activity against human cervix carcinoma (Hep2c), human rhabdomyosarcoma, and murine fibroblast (L2BO) cells, with the highest growth inhibition effect seen in yarrow infusion [69] (Table 5). Another study found that kombucha made from lemon balm had a growth-promoting effect at low concentrations on MCF-7 cells (ranging under 10 µg/mL) and HeLa cells (1.95–30 µg/mL) but at higher concentrations (500 µg/mL), it demonstrated a growth-inhibitory effect on MCF-7 cells, though not on HeLa cells. This can be explained by the hormesis of substances contained in kombucha [112].

Previous studies showed that kombucha had a stronger growth-inhibiting effect on cancer cells such as HCT-116 cells than its unfermented counterpart [47,121]. However, some studies suggested that kombucha may not be toxic to certain cancer cell lines such as human ovarian (OVCAR) and human colon (HT-29) cancer cells [113,121]. Moreover, African mustard (*Brassica tournefortii* Gouan) kombucha fermentation might not improve cytotoxicity on MCF-7 cells when compared to unfermented tea [72] (Table 5). These conflicting findings emphasize the need for further research in animal models and clinical studies to fully understand the anticancer properties and mechanisms of kombucha.

### 3.5. Antidiabetic Activities

One of the major epidemic health issues of the twenty-first century is diabetes mellitus (DM), which currently affects an estimated 463 million people globally and is projected to increase to 578 million in 2030 and 700 million in 2045 [122]. DM is a chronic metabolic disorder characterized by an increase in plasma glucose level (hyperglycemia) due to deficiency in insulin action or secretion and can be influenced by genetics, age, or lifestyle factors (e.g., diet habit, and physical activities). DM can be mainly divided into two types including type 1 DM (T1DM) and type 2 DM (T2DM). T1DM is known as insulin-dependent diabetes which is caused by autoimmune disorder that damages β-cells, insulin-producing cells in the pancreas, and results in reduced or no insulin production, whereas T2DM is the most common form and accounts for roughly 90% of DM cases involved in insulin resistance (IR) due to the cells poorly responding to the effect of insulin and, combined with relative insulin deficiency, leads to the impairment of glucose usage in target cells [123]. Hyperglycemia consequently causes macrovascular complications including cardiovascular diseases (e.g., cardiomyopathy, arrhythmias, and peripheral artery disease) and microvascular complications including retinopathy, lower extremity amputations, and end-stage renal disease, as well as being frequently linked to other pathological conditions such as infections, cancer, depression, and dementia that lead to chronic morbidities and mortality in diabetic patients [124].

Since DM is a complex disorder, attempts to manage DM in patients is a challenge. Several groups of synthetic drugs in conventional medicine have been developed for DM treatment, particularly for T2DM, such as insulin secretagogues, biguanides, and insulin sensitizers [125]. However, existing synthetic drugs have certain limitations and side effects in diabetic patients. Natural agents, such as medicinal plants, are now garnering more attention as alternatives for the management of DM. Kombucha made from tea or non-tea substrates, is a functional food that has shown outstanding beneficial health effects on DM (Table 6). Evidence has shown that kombucha prepared from green tea was able to prevent weight loss, which is one of clinical symptoms of T1DM, in diabetic rats [126]. The antihyperglycemic potential of kombucha has thus far been proven in a number of studies using diabetic animal models. Both healthy and alloxan-induced diabetic mice had a considerable reduction in blood sugar levels (BSL) after repeatedly consuming tea kombucha at 1.71 mL/kg for 3 days [127]. After 12 weeks of treatment, kombucha also decreased the glucose levels of obese mice to a level comparable to healthy mice [67]. Furthermore, snake fruit kombucha also significantly reduced fasting plasma glucose levels by 67 to 76%, enhanced Langerhans Island structure, and increased the number of pancreatic β-cells in diabetic rats at a rate comparable to the antidiabetic drug metformin [77]. Furthermore, it was previously found that after 45 days of daily kombucha administration, the level of glycated hemoglobin (HbA_1C_), an indicator of individual blood glucose level, which increased in streptozotocin-induced diabetic rats, dropped almost to normal levels, while the levels of indicators of improvement of DM including plasma insulin, hemoglobin, and tissue glycogen increased [128].

Kombucha can exert its antidiabetic effect by altering enzyme activity in glucose regulatory pathways, such as glycolysis and gluconeogenesis. It was previously demonstrated that plasma and pancreas α-amylase were reduced in alloxan-induced diabetic rats that were treated with black tea kombucha and these effects were better when compared to the effects of black tea [129]. Besides α-amylase, oak kombucha also showed potent in vitro inhibitory activity on α-glucosidase. In this sense, inhibition of these enzymes can delay starch hydrolysis and decrease postprandial plasma glucose levels, which could be beneficial for DM treatment [67]. In addition, kombucha from Chinese black tea, oolong tea, green tea, and Sri Lankan black tea exhibited inhibitory effects on both α-amylase and α-glucosidase [53]. Both enzymes were found in the small intestinal brush border and played an important role in the breakdown of carbohydrates. The α-amylase inhibitory activities in terms of the IC_50_ values ranged between 18.5 and 138.4 μg/mL, while the α-glucosidase inhibitory activities in terms of the IC_50_ values ranged between 69.3 and 158.7 μg/mL for all four kombucha samples [53] (Table 6). These enzymatic inhibitory activities led to the inhibition of starch hydrolysis, resulting in the attenuation of glucose absorption from the gastrointestinal tract. Furthermore, kombucha restored the activities of the enzymes crucial for glycolysis and gluconeogenesis, including glucose-6-phosphatase, fructose-1,6-bisphosphatase, and hexokinase, to a level close to normal in diabetic rats [128].

Recently, it was shown that gut microbiota can play a crucial role in the onset of T2DM. Imbalance in the number of pathogenic and beneficial bacteria can damage the intestinal mucosal barrier which can lead to β-cell damage. However, kombucha can alter the composition of gut microbiota by increasing firmicutes (healthy bacteria) while reducing proteobacteria (unhealthy bacteria) in diabetic mice. In particular, kombucha boosted the population of short-chain fatty acid (SCFAs)-producing bacteria which participate in SCFA production, which improved β-cell function in T2DM [130].

In addition to its antidiabetic properties, kombucha is now being studied for its protective effects against the secondary complications of DM, such as nephropathy. Kombucha brought the levels of urea, uric acid, and creatinine in diabetic rats with nephropathy nearly back to normal levels compared to control rats. By restoring the glomerular and tubular structures, kombucha lessened kidney damage caused by DM [131]. Kombucha can also suppress oxidative stress-mediated tissue and organ damage, which play a role in the development of diabetic complications through the modulation of antioxidant enzyme activities and oxidative stress-related parameters in alloxan-induced diabetic rats [46].

Several biochemical ingredients and properties of kombucha, such as bioactive compounds, pH and microbial community structure, can change during the course of fermentation [18]. Kombucha derived from different substrates, such as snake fruit with five cultivars, could show a difference in physicochemical characteristics that could affect their bioactivities [76]. The favorable effects of kombucha on the prevention of DM and other diseases related to DM might be attributed to its active ingredients, especially phenolic compounds which form during the fermentation process (Table 6). The inhibitory effect of kombucha on enzyme activity involved in glucose metabolism including α-amylase and α-glucosidase was reviewed in many studies [7]. Interestingly, the inhibition of porcine pancreatic α-amylase activity is primarily affected by phenolic compounds, particularly catechins such as EGCG, GCG, and ECG and that inhibition potency increased during fermentation [132]. Furthermore, polyphenols such as quercetin and epicatechin have been proposed as contributors to the protection of pancreatic β-cell damage in diabetic rats [129,133].

### 3.6. Antihypertensive Effects

Hypertension is the “silent disease” which affects people all over the world and often increases the risk of other serious health problems such as cardiovascular diseases (e.g., aneurysms, myocardial infarction, stroke and heart failure). The renin-angiotensin system (RAS) has long been recognized as playing a crucial role in controlling blood pressure and fluid balance in the body and that RAS overstimulation considerably exacerbates the condition of high blood pressure [134,135]. Angiotensin II (Ang II) and angiotensin-converting enzyme (ACE) are two primary components of RAS that contribute to hypertensive effects. ACE is responsible for Ang II production by encouraging the conversion of angiotensin I into angiotensin II, so that Ang II subsequently causes vasoconstriction [134,135]. Thus, the use of ACE inhibitors (ACEI) has become one of the major focuses for the alleviation of hypertension and is considered one of the first-line drugs for treatment of hypertension [136]. ACEIs are synthetic compounds, the use of which have adverse effects such as dry cough, angioedema, and hyperkalemia and ACEIs should be not used in pregnant women. Due to these adverse effects, many scientists have explored bioactive compounds from natural sources that possess ACE inhibitory properties for the potential treatment of hypertension.

Most previous studies have reported that kombucha samples from a variety of herbs exhibited antihypertensive effects primarily through the inhibition of ACE activity (Table 7). For example, kombucha-fermented milk showed the highest ACE inhibitory effect on day 14 but has a minimal effect on day 7 of fermentation. Moreover, kombucha-fermented milk demonstrated the strongest efficacy on ACE inhibition (IC_50_ = 0.33 mg/mL) when compared to regular fermented milk that was probiotic and yoghurt-based (IC_50_ = 0.25 mg/mL) [137]. In a similar manner, after 72 h of fermentation, ultrafiltered extracts of kombucha-fermented milk had the highest ACE inhibitory activity with 93% ACE inhibition when compared to lactic acid bacteria-fermented milk [138]. It was demonstrated that bioactive peptides derived from fermented milk (VAPFPEVFGK, LVYPFPGPLH, and FVAPEPFVFGKEK) have antihypertensive effects. For this reason, the blood pressure-lowering action of kombucha fermented milk may be due to ACE inhibitory peptides that are enzymatically produced from the precursor protein during milk fermentation [139].

Based on the expanded uses of alternative substrates, rather than using traditional black or green tea substrates for kombucha production, several studies have compared the ACE inhibitory effect of different substrates and conditions. For instance, this study compared the ACE inhibitory effect of kombucha prepared with traditional substrates versus six alternative herbal substrates, including winter savory (*Satureja montana*), peppermint (*Mentha×piperita*), stinging nettle (*Urtica dioica*), wild thyme (*Thymus serpyllum*), elderberry (*Sambucus nigra*), and quince (*Cydonia oblonga*) [64] (Table 7). As a result, all substrates exhibited ACE inhibitory properties with IC_50_ values ranging from 8.03 to 140.81 mL in kombucha samples. Interestingly, due to its high phenolic content, green- and black tea kombucha has demonstrated a greater inhibitory ACE impact than herbal kombucha samples [64].

Furthermore, the herbs *E. camaldulensis* and *L. glaucescens*, both fermented (kombucha) and unfermented (infusion), have been found to have inhibitory effects on ACE. The IC_50_ of kombucha was shown to be higher compared with unfermented beverages of the plant itself. However, compared with captopril, a commercial ACE inhibitor, both infusion and fermented beverages demonstrated lower IC_50_ values, showing their effectiveness as potential alternative hypertensive agents [140] (Table 7). Gamboa-Gómez and colleagues proposed that phenolic compounds and flavonoids contained in kombucha samples might have antihypertensive properties [140]. Nonetheless, this study found that catechin, which is one of major phenolics, did not show ACE inhibitory activity [140]. Interestingly, our previous study demonstrated that quercetin can inhibit the RAS pathway by inhibiting ACE activity and reducing ACE synthesis in HEK-293 cells [141]. Therefore, the further identification of phytochemicals corresponding to the ACE inhibitory activities of each kombucha sample is worthy of investigation.

Kombucha also has a protective effect against heart diseases. In rats with myocardial infarctions, kombucha showed a protective role in membrane stabilization through the modulation of transmembrane protein activities, including Na^+^/K^+^ ATPase, Ca^2+^ ATPase, and Mg^2+^ ATPase, while unfermented black tea showed no change. Moreover, kombucha reduced cardiomyocyte destruction, which further preserved the normal heart tissue architecture [142]. However, this study did not show the effects of any antihypertensive properties. Collectively, it should be noted that the measurement of ACE activity came from an in vitro study. The animal models of hypertension should be performed to confirm the antihypertensive effects of these kombucha beverages.

### 3.7. Antihyperlipidemic Effects

Hyperlipidemia includes hypercholesterolemia and hypertriglyceridemia and is a metabolic disorder defined by the elevation of lipids, specifically, cholesterol, triglycerides, and lipoproteins, in the blood [143]. An abnormal lipid profile, characterized by a high level of triglycerides (TG), total cholesterol (TC), and low-density lipoprotein cholesterol (LDL-C) as well as a low level of high-density lipoprotein cholesterol (HDL-C), is a well-known feature of hyperlipidemia and is recognized as a strong risk factor for cardiovascular diseases (CVD) such as heart, stroke, coronary heart diseases (CHD), and atherosclerosis [143,144]. Hence, the modulation of abnormal lipid metabolism might provide beneficial outcomes in the treatment of hyperlipidemia.

The consumption of kombucha demonstrates a cholesterol-lowering effect and an improvement of lipid profiles, providing protection against hyperlipidemia in both healthy and disease animal models (Table 8). The addition of kombucha to drinking water at various doses (10–25%) decreased the total cholesterol and LDL-C while also increasing HDL-C levels in duck blood, with the highest dose (25%) showing the greatest impact on the lipid profile [145]. Similarly, this improvement in the lipid profile was also found in rats treated with black tea and skim-milk kombucha [146] (Table 8). A high-fat diet is one of the most important factors contributing to hyperlipidemia. The consumption of a cholesterol-rich diet markedly increases levels of triglycerides, LDL, and cholesterol in rabbits and rodents. However, kombucha has been shown to reverse these parameters and increase HDL levels [51,55,147]. Furthermore, fat accumulation in the artery wall, known as atherosclerotic plaque, that developed in rabbits fed a high cholesterol diet was reduced, as demonstrated by a reduction in plaque thickness and the filtration of inflammatory cells after kombucha consumption, suggesting the possible protective role of kombucha in hyperlipidemia associated with coronary artery diseases [148].

It should be noted that the cholesterol-lowering action of kombucha was linked to the presence of phenolic compounds such as catechins, which is probably limited cholesterol absorption in the small intestine, leading to a reduction in the amount of cholesterol that enters the bloodstream [20]. An inverse correlation between HDL and CHD has been addressed in numerous studies. The elevation of HDL aids in the transportation of cholesterol form serum to the liver, where it will subsequently be further removed from the body. As a result, HDL could potentially reduce the risk of CHD events [149].

DM is frequently accompanied by dyslipidemia, including abnormal lipid profiles (e.g., increased in LDL production and reduced in HDL synthesis), which is widely regarded as a major CVD risk factor [143,150]. Previous research has revealed that diabetic rats exhibit a poor lipid profile, but this was reversed after kombucha treatment [77,129]. Moreover, snake fruit kombucha proved to be more successful in improving the lipid profile than the antidiabetic drug metformin after 28 days of experimenting on diabetic rats [77]. An increase in the activity of lipases, enzymes that play a key role in the breakdown and absorption of lipids, was observed in the plasma and pancreas of diabetic rats [129]. Elevation in lipase activity, in turn, led to improved digestion and the absorption of lipids such as triglycerides and LDL-C, exacerbating the development of hyperlipidemia. However, kombucha (5 mL/kg per day) supplementation for 30 days dramatically reduced lipase activity, delaying the absorption of triglycerides and LDL-C as well as increasing the HDL-C level [129]. In vitro experiments have shown that polyphenols have the ability to inhibit pancreatic lipase activity. Therefore, it is possible that the inhibitory effect of kombucha on lipase activity can be attributed to the presence of polyphenols such as EGCG [151].

The effect of kombucha has also drawn attention to its antioxidant activity, which contributed to the prevention of lipid peroxidation, which is a situation where lipids are damaged by oxidative stress [20]. Kombucha given to rats on a high cholesterol diet was shown to considerably decrease the lipid peroxidation index in the liver and kidney to 55% and 44%, respectively [51]. As an oxidized LDL, it can damage the artery wall involved in the progression of atherosclerosis. However, it was previously revealed that kombucha offered LDL protection against oxidation in hyperlipidemic mice, which was related to the scavenging ability of polyphenols, highlighting the role of kombucha in protection against atherosclerosis [55].

## 4. Conclusions

For a long time, Kombucha beverages have been claimed to have various health-promoting and beneficial effects all over the world. Even though nowadays kombucha beverages are known worldwide, their biological properties are not quite understood. Their current strengths are known based on promising results that were obtained from in vitro studies and a few of animal studies, proving antioxidant, anti-inflammatory, antimicrobial, anticancer, antidiabetic, antihypertensive, and antihyperlipidemic effects, as well as other properties. Despite the potential health benefits associated with consuming kombucha beverages, there are some challenges that limit their use, and the comparable mechanisms of their active compounds and their role in physiological functions in the human body remain unclear.

Currently, there are insufficient data on kombucha beverages regarding the therapeutic treatment of diseases. In addition, it should be noted that results obtained from in vitro studies may significantly differ from those obtained from in vivo and clinical studies. Therefore, it is necessary to deepen in vivo and clinical trials to confirm the potential activities of kombucha beverages that have been found in numerous in vitro studies. Based on a diversity of bioactive compounds found in kombucha, as well as a variety of raw materials, further studies should focus on the purification of targeted bioactive compounds and the evaluation of their pharmacological activities. This will allow for a more reliable determination of the beneficial effects of kombucha beverages as “functional” foods.

## 5. Limitation

Kombucha beverages are a major source of bioactive compounds in tea, SCOBY, and raw materials. The type and number of these compounds change depending on several factors as mentioned above, affecting the composition of kombucha. Therefore, the biological activities of kombucha beverages regarding health-promoting properties should not be generalized since there are several factors that affect the components of kombucha; hence, the standardization of kombucha beverages is required to confirm their biological activities. The FDA has stated that when safely produced, kombucha beverages can be safe for human consumption. However, the adverse effects and toxicity associated with the excessive and prolonged consumption of kombucha beverages are still unclear. For this reason, to avoid adverse effects, the consumption of kombucha beverages is contraindicated in special populations, including pregnant women, infants, and children under the age of 4. Taken together, further research involving more in-clinical trials are required to validate and confirm the pharmacological effects, as well as the adverse effects and toxicity, of kombucha beverages.

## Figures and Tables

**Table 2:** Anti-inflammatory and immunomodulatory activities of kombucha tea and kombucha beverages made from a variety of raw materials.

Name of Substrates	Active Ingredients	Biological Assays	Findings	Ref.
**In vitro studies**				
Black tea	▪ Phenolic compounds: gallic acid, catechin, (-)-epicatechin, caffeine, *p*-coumaric acid, ferulic acid, rutin hydrate, taxifolin, etc.	▪ 5-LOX enzyme inhibition assay	▪ Anti-inflammatory activity of black tea kombucha was higher than unfermented tea. ▪ Ethyl acetate fractions from kombucha showed highest 5-LOX inhibitory activity ▪ Aqueous fractions had less inhibitory effects against 15-LOX.	[47]
Black tea, white oak leaves (*Quercus resinosa*, *Q. arizonica*, *Q. convallata*)	▪ Phenolic compounds: gallic acid, epicatechin, gallocatechin, catechin, gallocatechin gallate, etc.	▪ LPS induced-NO and cytokines (IL-6, and TNF-α) production in RAW264.7 macrophages	▪ All kombuchas decreased NO production in LPS-induced macrophages. ▪ White oak kombuchas showed significant inhibitory effect on TNF-α production in LPS-induced macrophages. ▪ *Q. resinosa* and *Q. convallata* kombuchas significantly suppressed IL-6 production.	[66]
Mushrooms (*Coriolus versicolor* and *Lentinus edodes*)	▪ Polysaccharides ▪ Phenols ▪ Flavonoids	▪ MTT assay to determine metabolic activity ▪ Phytohemagglutinin (PHA) stimulated peripheral blood mononuclear cell (PBMC) cultures: cytokines analysis	▪ Immunomodulatory effects of the mushroom kombuchas were related to their polysaccharide composition. ▪ Polysaccharide extracts from mushroom kombuchas downregulated anti-inflammatory cytokines (IL-4, IL-5 and IL-10) and upregulate of pro-inflammatory cytokines (TNF-α, IL-6, and IL-8) in PHA-stimulated PBMC cultures.	[95]
Green tea	▪ Organic acids: glucuronic acid, acetic acid, formic acid, etc.	▪ LPS induced-NO and cytokines (IL-1α, IL-6, and TNF-α) production in RAW264.7 cells	▪ Green tea kombucha could reduce NO, IL-1α, IL-6, and TNF-α production in LPS-treated RAW264.7 cells.	[96]
**In vivo studies**				
Black tea	▪ Not determined	▪ Myelin oligodendrocyte glycoprotein (MOG_35–55_)-induced experimental autoimmune encephalomyelitis (EAE) C57BL/6 mice ▪ Measurement of T-helper-related cytokines in splenocytes and lymph node cells ▪ Measurement of NO in blood and spinal cord	▪ Treatment of the EAE-induced mice with kombucha attenuated EAE clinical symptoms. ▪ Kombucha increased production of IL-4 and TGF-β and reduced IL-17, IFN-γ, and NO synthesis.	[89]
Green tea	▪ Organic acids: acetic acid, gluconic acid, and glucuronic acid ▪ Phenolic compounds: catechins, EGCG, ECG, EGC, EC, etc.	▪ LPS-induced sepsis in mice ▪ Histopathological analysis ▪ Measurement of serum inflammatory cytokine levels (IL-1β, IL-6, and TNF-α) ▪ Measurement of cytokine mRNA expression in lung tissues	▪ Survival rate and thermoregulation of LPS-treated mice increases by 40% when treated with kombucha. ▪ Treatment with kombucha resulted in reduction of TNF-α, IL-1β, and IL-6 levels in LPS-challenged mice and inhibition of histopathological damage in LPS-induced sepsis mice.	[97]
Black tea	▪ Phenolic compounds: theaflavin, thearubigins	▪ Indomethacin-induced stomach ulceration in Swiss albino mice ▪ Histopathological and biochemical parameter analysis ▪ Mucin assay ▪ Non-protein thiol (NP-TSH) assay	▪ Increased mucin content; decreased macroscopic damage scores (MDS), gastric acid secretion, TBARS and non-protein thiol (NP-TSH) levels in the gastric tissues.	[99]
Black tea	▪ Not determined	▪ Myelin oligodendrocyte glycoprotein (MOG_35–55_)-induced experimental autoimmune encephalomyelitis (EAE) mice ▪ Histopathological analysis of CNS by LFB and H&E staining on brains and cerebellums ▪ Measurement of NO and TNF-α in serum	▪ Kombucha tea decreased EAE incidence and delayed EAE onset and disease progression of the treated mice ▪ Demyelination, neuronal degeneration, and infiltration of inflammatory cells in brains and cerebellums of kombucha tea-treated mice were less than control mice. ▪ Treatment with kombucha decreased in-serum NO and TNF-α levels.	[100]
Black tea and arabica coffee	▪ Phenolic compounds: flavonoids, tannins	▪ In vivo immunomodulatory activity (*Salmonella typhi*-infected Balb-C mice) ▪ Immunomodulatory activity assays	▪ Black tea and arabica leaf kombuchas enhanced adaptive immune response due to improvement in CD4+, CD8+, CD4+IFN-γ, CD4+TNFα, CD8+IFN-γ, and CD8+TNFα. ▪ Black tea kombucha had higher immunomodulatory ability than arabica leaf kombucha.	[101]
Black tea and turmeric	▪ Phenolic compounds	▪ In vivo immunomodulatory activity (*Salmonella typhi*-infected Balb-C mice) ▪ Immunomodulatory activity assays	▪ Turmeric and black kombucha enhanced the adaptive immune response (indicated by increases in CD4^+^, TNFα, IFN-γ) and innate immune response (evidenced by decrease of CD68^+^ IL-6^+^).	[102]

**Table 3:** Antibacterial activities of kombucha tea and kombucha beverages made from a variety of raw materials.

Name of Substrates	Active Ingredients	Biological Assays	Findings	Ref.
**In vitro studies**				
Soy whey	▪ Phenolic compounds ▪ Organic acids: glucuronic acid, formic acid, acetic acid, and citric acid	▪ Agar well diffusion (fermented soy whey, unfermented soy whey, neutralized kombucha, heat-denatured kombucha)	▪ Soy whey kombucha showed higher antibacterial activity against *S. aureus*, *B. subtilis* and *E. coli* than neutralized kombucha, heat-denatured kombucha, and unfermented soy whey. ▪ Antibacterial activity of kombuchas based on organic acids and other compounds such as metabolites.	[15]
Black and green teas	▪ Phenolic compounds: theaflavin, thearubigin, catechin, gallocatechin 3-*O*-gallate, etc. ▪ Organic acids: 5-*O*-galloyquinic acid, ▪ 4-coumaroylquinic acid derivatives, etc.	▪ Broth microdilution ▪ (black tea and green tea kombuchas)	▪ Green tea kombucha showed antibacterial activity against *S. aureus*, *S. Typhimurium*, *E. coli*, and *L. monocytogenes*. ▪ Black tea kombucha inhibited growth of *S. aureus* and *L. monocytogenes*. ▪ Green tea kombucha had higher antibacterial activity than black tea kombucha.	[49]
Black, green, and oolong teas	▪ Phenolic compounds ▪ Organic acids: acetic acid, gluconic acid, D-saccharic acid 1,4-lactone, glucuronic acid, ascorbic acid	▪ Diffusion method (fermented infusion, unfermented infusion, neutralized kombucha, heat-denatured kombucha)	▪ Tea kombuchas showed antibacterial activity against *E. coli*, *S. dysenteriae*, *S. typhi*, and *V. cholerae.* ▪ Unfermented tea and neutralized fermented tea had no antibacterial activity against all tested bacteria. ▪ Heat-denatured kombucha by boiling caused a greater decrease in antibacterial activity than autoclaving.	[52]
Yerba-maté (*Ilex paraguariensis*)	▪ Phenolic compounds ▪ Organic acid: acetic acid	▪ Agar disc diffusion	▪ Yerba-maté kombucha exhibited antibacterial activity against *E. coli* and *S. aureus.*	[58]
Lemon balm (*Melissa officinalis*)	▪ Phenolic compounds: rosmarinic acid, caffeic acid, quercetin, chlorogenic acid, ferulic acid ▪ Organic acids: acetic acid	▪ Agar well diffusion (fermented infusion, unfermented infusion, acidified infusion, neutralized kombucha, heat-denatured kombucha)	▪ Lemon balm kombucha exhibited antibacterial activity against *P. aeruginosa*, *Proteus *sp., *E. coli*, *C. freundii*, *Enterobacter cloacae*, *Salmonella* sp., *S. saprophyticus*, *S. equorum*, *Bacillus* sp., *L. monocytogenes*, and *L. innocua.* ▪ Neutralized kombucha had lower antibacterial activity than untreated kombucha.	[61]
Yarrow (*Achillea millefolium*)	▪ Phenolic compounds ▪ Organic acids: xalic acid, formic acid, acetic acid, lactic acid, etc.	▪ Microdilution method in 96-multi-well microtiter plates	▪ Kombuchas showed antibacterial activity against *S. aureus*, *K. pneumoniae*, *E. coli*, *P. vulgaris*, *P. mirabilis*, and *B. subtilis* (MIC = 9.77 − 312.50 µg/mL).	[69]
Black tea and garlic	▪ Phenolic compounds	▪ Agar disc diffusion (fresh garlic extract, fermented garlic extract, fresh kombucha, kombucha after fermentation with garlic)	▪ Fresh garlic and kombucha-fermented garlic extracts showed antibacterial activity against *S. saprophyticus*, *S. aureus*, *S. epidermidis*, *B. stearothermophilus*, *S. typhimurium*, *E. coli*, and *P. aeruginosa* but less than fresh garlic extract. ▪ Fresh garlic and kombucha-fermented garlic extracts had strong inhibitory effect against Gram-positive bacteria.	[70]
Apple varieties (Anna, Fuji, Granny Smith, Manalagi, Red Delicious, Rome Beauty, Royal Gala)	▪ Phenolic compounds ▪ Organic acid: acetic acid	▪ Agar well diffusion	▪ All kombuchas exhibited antibacterial activity against *E. coli* and *S. aureus*. ▪ Antibacterial activity of kombuchas increased during the fermentation process due to organic acid production.	[75]
Snake fruit (*Salacca zalacca*)	▪ Phenolic compounds ▪ Organic acids: acetic acid	▪ Agar well diffusion	▪ Snake fruit kombucha showed higher antibacterial activity against *E. coli* and *S. aureus* than sugared snake fruit juice before fermentation.	[76]
Cactus pear	▪ Phenolic compounds and betalains ▪ Organic acid: acetic acid	▪ Agar well diffusion (fermented juice, unfermented juice, neutralized fermented juice)	▪ Kombucha showed antibacterial activity against *S. epidermidis*, *S. aureus*, *B. cereus*, *E. faecalis*, *K. pneumoniae*, *E. coli*, and *P. aeruginosa.* ▪ Unfermented juice and neutralized fermented juice had no antibacterial activity against all tested bacteria.	[78]
Red grape	▪ Phenolic compounds and anthocyanins	▪ Agar well diffusion (fermented juice, unfermented juice, neutralized fermented juice)	▪ Fermented grape juice showed antibacterial activity against *S. epidermidis*, *S. aureus*, *B. cereus*, *E. faecalis*, *K. pneumoniae*, *E. coli*, and *P. aeruginosa.* ▪ Neutralized fermented juice showed antibacterial activity against *S. aureus* but less than fermented juice.	[79]
Green tea	▪ Organic acids: glucuronic acid, acetic acid, formic acid, lactic acid, citric acid	▪ Agar well diffusion (kombucha fermented for 0, 1, 4, and 7 days, unfermented infusion, heat-denatured kombucha)	▪ Kombucha showed an increase in antibacterial activity against *S. aureus*, *E. coli*, and *S. enterica* during fermentation process. ▪ Heat-denatured kombucha had lower antibacterial activity against *E. coli*, and *S. enterica* than untreated kombucha.	[96]
Black tea	▪ Heterocyclic compounds ▪ Lactones ▪ Antibiotics ▪ Unsaturated fatty acid ▪ Alkaloids: caffeine	▪ Agar well diffusion (fermented infusion, unfermented infusion, neutralized kombucha, heat-denatured kombucha)	▪ Kombucha showed antibacterial activity against *S. aureus*, *B. cereus*, *L. monocytogenes*, *E. coli*, and *S. typhimurium* higher than heat-denatured kombucha and neutralized kombucha. ▪ Unfermented tea had no antibacterial activity against most tested bacteria.	[106]
Black and green teas	▪ Organic acids	▪ Agar well diffusion (fermented infusion, unfermented infusion, acidified infusion, neutralized kombucha, heat-denatured kombucha)	▪ Kombucha showed antibacterial activity against *S. aureus*, *S. epidermidis*, *M. luteus*, *L. monocytogenes*, *E. coli*, *P. aeruginosa*, and *S. typhimurium.* ▪ All neutralized kombuchas had lower antibacterial activity than untreated kombucha. ▪ Heat-denatured kombuchas showed a slight decrease in antibacterial activity against most bacteria.	[107]
Black and green tea, lemon verbena (*Lippia citriodora*), and peppermint (*Mentha piperita*)	▪ Organic acids: acetic acid	▪ Agar well diffusion	▪ All kombucha samples showed the highest antibacterial activity against *E. coli*, *S. aureus*, *B. cereus*, and *S. dysenteriae.* ▪ Black tea kombucha showed the highest antibacterial activity against *B. cereus.* ▪ Kombuchas prepared from green tea and peppermint showed the highest antibacterial activity against *B. cereus.* ▪ Peppermint and lemon verbena kombuchas exhibited the highest antibacterial activity against *E. coli* and *S. dysenteriae*.	[108]
Black tea	▪ Phenolic compounds ▪ Organic acids: acetic acid, tartaric acid, citric acid	▪ Agar disc diffusion (kombucha tea, unfermented sweet tea)	▪ Kombucha showed antibacterial activity against *E. coli*, and *H. influenzae* with larger inhibition zone than unfermented sweet tea.	[109]
Black, green, oolong, and mulberry teas	▪ Organic acids	▪ Agar disc diffusion (fermented kombuchas, unfermented infusion, neutralized kombucha)	▪ Black tea kombucha showed an increase in antibacterial activity against *V. cholerae*, *S. typhi*, *P. aeruginosa.* ▪ Kombuchas made from green, oolong, and mulberry teas showed an increase in antibacterial activity against *V. cholerae*. ▪ Unfermented tea and neutralized fermented tea had no antibacterial activity.	[110]
Green tea and spices (cinnamon, cardamom, and Shirazi thyme)	▪ Phenolic compounds ▪ Organic acids: acetic acid, glucuronic acid, lactic acid, citric acid, tartaric acid, oxalic acid, malic acid	▪ Broth microdilution (green tea kombucha, cinnamon-flavored kombucha, cardamom-flavored kombucha, Shirazi thyme-flavored kombucha)	▪ Green tea kombucha and spice-flavored kombuchas showed antibacterial activity against *S. aureus*, *B. cereus*, *E. coli*, and *S. typhimurium*. ▪ Cinnamon-flavored kombucha showed the best antibacterial activity against *E. coli* and *S. typhimurium.* ▪ Increasing cinnamon concentration in kombucha tended to decrease the MIC values against all bacteria.	[111]
Lemon balm (*Melissa officinalis*)	▪ Organic acid: acetic acid	▪ Agar well diffusion (fermented infusion, unfermented infusion, acidified infusion, neutralized kombucha, heat-denatured kombucha)	▪ Lemon balm kombucha exhibited antibacterial activity against *P. aeruginosa*, *P. mirabilis*, *E. coli*, *C. freundii*, *Erwinia carotovora*, *S. enteritidis*, and *S. aureus*. ▪ Heat-denatured kombucha had no significant antibacterial activity compared to kombucha sample.	[112]
Thyme (*Thymus vulgaris*), lemon verbena (*Lippia citriodora*), rosemary (*Rosmarinus officinalis*), fennel (*Foeniculum vulgare*), and peppermint (*Mentha piperita*)	▪ Organic acids	▪ Agar well diffusion (fermented infusion, unfermented infusion, acidified infusion, neutralized kombucha, heat-denatured kombucha)	▪ Kombuchas except for *T. vulgaris* kombuchas showed antibacterial activity against *S. epidermidis*, *S. aureus*, *L.* monocytogenes, *M. luteus*, *E. coli*, *P. aeruginosa*, and *S. typhimurium.* ▪ Acidity is important for antibacterial activity. ▪ All neutralized kombuchas had lower antibacterial activity than untreated kombucha.	[113]
Beet (*Beta Vulgaris*)	▪ Phenolic compounds ▪ Organic acids: acetic acid, lactic acid	▪ Agar disc diffusion (kombuchas, unfermented beet filtrate)	▪ Beet kombucha showed antibacterial activity against *E. coli* with an activity index of 53.57 % when using streptomycin sulfate as a positive control.	[114]

**Table 5:** Anticancer activities of kombucha tea and kombucha beverages made from a variety of raw materials.

Name of Substrates	Active Ingredients	Biological Assays	Findings	Ref.
**In vitro studies**				
Black tea	▪ Phenolic compounds: gallic acid, caffeic acid, catechin, chlorogenic acid, epicatechin, etc. ▪ Alkaloids: caffeine, theobromine	▪ Cytotoxic activity by MTT assay in cancer cells (MCF-7 and HCT-116)	▪ The highest percentage inhibition of cell proliferation was found in ethyl acetate fraction of kombucha tea in MCF-7 and HCT-116. ▪ Kombucha fermentation has more antiproliferative activity than unfermented samples.	[47]
Yarrow (*Achillea millefolium* L.)	▪ Phenolic compounds ▪ Flavonoids ▪ Organic acids ▪ Vitamin C	▪ Antiproliferative activity by MTT assay in cancer cells (RD, Hep2C) and normal cells (L2OB) ▪ Wound-healing assay ▪ mRNA expression	▪ All samples (infusion and fermented kombucha) exhibited antiproliferative activity. ▪ Infusions were most effective against Hep2C while kombucha had the highest activity against RD cells. ▪ Yarrow kombucha beverages showed lower antiproliferative activity compared to infusion products.	[69]
African mustard (*Brassica tournefortii*) leaves	▪ Phenolic compounds ▪ 2-Hydroxy-3-methylbutyric acid ▪ Glycerol ▪ Butanedioic acid	▪ Cytotoxic activity by MTT assay in cancer cells (MCF-7)	▪ Ethyl acetate fraction of unfermented African mustard has the highest cyotoxic activity compared other fractions (45% inhibition).	[72]
Lemon balm (*Melissa officinalis* L.)	▪ Organic acid: acetic acid	▪ Cell growth by sulforhodamine B assay in cancer cells (HeLa, MCF7, HT-29)	▪ Kombucha from lemon balm tea exhibited antiproliferative activity against cancer cells at high concentration (500 mg/mL). ▪ Lemon balm kombucha showed higher antiproliferative activity than traditional kombucha tea.	[112]
Black tea	▪ Dimethyl 2-(2-hydroxy-2-methoxypropylidine)malonate ▪ Vitexin	▪ Antiproliferative activity by MTT assay in cancer cells (A549, U2OS, 786-O) ▪ Cell invasion and migration assay ▪ MMP activity by gelatin-zymography protease assay	▪ Ethyl acetate fraction of kombucha (100 mg/mL) exhibited antiproliferative effects on U2OS and 786-O cells. ▪ Ethyl acetate fraction reduced cell invasion and motility in A549, U2OS and 786-O cells. ▪ Ethyl acetate fraction inhibited MMP-2 and MMP-9 activities in 786-O cells, and MMP-2 activity in A549 cells.	[118]
Black tea	▪ Not determined	▪ Cell viability by MTT assay in prostate cancer cells (PC-3) ▪ Wound-healing assay ▪ mRNA expression	▪ Kombucha decreased PC-3 survival with IC_50_ values of 400 mg/mL. ▪ Kombucha inhibited migration of PC-3 cells. ▪ Kombucha downregulated mRNA expression of HIF-1α, VEGF, IL-8, and COX-2, thereby inhibiting angiogenesis.	[119]
Green tea and black tea	▪ Not determined	▪ Cell viability by MTT assay in cancer cells (A549, Hep-2)	▪ Kombucha green tea exhibited antiproliferative effects on A549 and Hep-2 with IC_50_ values of 250 and 200 mg/mL, respectively. ▪ Kombucha black tea exhibited antiproliferative effects on Hep-2 with IC_50_ values of 386 mg/mL and had no effect against A549 cancer cells.	[120]

**Table 6:** Antidiabetic effects of kombucha tea and kombucha beverages made from a variety of raw materials.

Name of Substrates	Active Ingredients	Biological Assays	Findings	Ref.
**In vitro studies**				
Soymilk	▪ Isoflavones: daidzin, glycitin, genistin, etc. ▪ Phenolic acids: ferulic acid, chlorogenic acid ▪ Vitamins: riboflavin, ascorbic acid, α-tocopherol)	▪ α-amylase inhibitory activity ▪ α-glucosidase inhibitory activity	▪ α-amylase and α-glucosidase inhibitory activities significantly increased during fermentation.	[14]
Chinese black tea, oolong tea, green tea and Sri Lankan black tea	▪ Epicatechin isomers: epicatechin, epicatechin-3-gallate, epigallocatechin, etc. ▪ Catechin ▪ Gallic acid	▪ α-amylase inhibitory activity ▪ α-glucosidase inhibitory activity	▪ All four kombuchas exhibited a-amylase and α-glucosidase inhibitory activities. ▪ Sri Lankan black tea has the highest α-amylase and α-glucosidase inhibitory activities.	[53]
Oak leaves [*Quercus convallata* (QC) and *Quercus arizonica* (QA)]	▪ Hydroxybenzoic acids ▪ Hydroxycinnamic acids ▪ Flavan-3-ols ▪ Flavonols ▪ Flavanone ▪ Dihydrochalcone	▪ α-amylase inhibitory activity ▪ α-glucosidase inhibitory activity	▪ QA kombucha and QC kombucha had lower α-amylase inhibitory activities than acarbose. ▪ QA kombucha and QC kombucha exhibited higher α-glucosidase activities than acarbose at all concentrations.	[67]
**In vivo studies**				
Oak leaves [*Quercus convallata* (QC) and *Quercus arizonica* (QA)]	▪ Hydroxybenzoic acids ▪ Hydroxycinnamic acids ▪ Flavan-3-ols ▪ Flavonols ▪ Flavanone ▪ Dihydrochalcone	▪ Female C57BL/6 mice having high- saturated fat and -fructose diet-induced obesity were treated with 31 mg of sample per kg bw for 3 months ▪ Oral glucose tolerance test (OGTT) ▪ Fasting plasma glucose (FPG) levels ▪ Insulin resistance determined by the product of triglycerides and glucose (TyG)	▪ After OGTT, group treated with oak leaf kombucha showed lower plasma glucose levels compared with obesity group (reduced 18%). ▪ Mice treated with kombucha from oak leaves exhibited lower levels of FPG than both healthy and obese control mice (reduced 52–66%). ▪ QA and QC kombucha-treated mice showed significant reductions in TyG index (reduced 5.9–7.5%).	[67]
Snake fruit (*Salak Suwaru* cultiva) and black tea	▪ Phenolic compounds	▪ Male Wistar rats were injected with streptozotocin to induce DM. DM rats were orally administered with snake fruit kombucha (5 mL/kg bw/day) or metformin (45 mg/kg bw/day) for 28 days. ▪ Fasting plasma glucose (FPG) levels ▪ Immunohistochemical staining of pancreatic tissues	▪ Snake fruit kombucha and black tea kombucha reduced fasting plasma glucose levels (reduction of 67–76%) and improved oxidative stress and lipid profiles. ▪ DM rat group with metformin had a high number of pancreatic β-cells, which is not significantly different from snake fruit kombucha. ▪ DM rat group with snake fruit kombucha exhibited higher number of β-cells than black tea kombucha.	[77]
Black tea	▪ Not determined	▪ Alloxan-induced diabetic rats were given black tea or kombucha tea at 5 mL per kg bw for 30 days ▪ Plasma glucose level ▪ Plasma and pancreas α-amylase activity	▪ Black tea and kombucha tea decreased plasma glucose levels and inhibited α-amylase activity in plasma and pancreas. ▪ Compared to black tea, kombucha tea was a better inhibitor of α-amylase activity and a better suppressor of increased plasma glucose levels.	[129]

**Table 7:** Antihypertensive effects of kombucha tea and kombucha beverages made from a variety of raw materials.

Name of Substrates	Active Ingredients	Biological Assays	Findings	Ref.
**In vitro studies**				
Black tea, green tea, winter savory (*Satureja montana*), peppermint (*Mentha × piperita*), stinging nettle (*Urtica dioica*), wild thyme (*Thymus serpyllum*), elderberry (*Sambucus nigra*), and quince (*Cydonia oblonga*)	▪ Organic acids ▪ Phenolic compounds ▪ Flavonoids	▪ ACE inhibitory activity	▪ All kombucha samples inhibited ACE activity with the IC_50_ values in range from 8.03–140.81 mL of beverage. ▪ Black tea and green tea kombuchas had high activity. ▪ Kombucha from winter savory, peppermint, wild thyme, and quince had lower ACE inhibitory activity compared to tea.	[64]
Milk	▪ Total proteins	▪ ACE inhibitory activity of water-soluble extracts from kombucha-fermented milk	▪ Milk kombucha had the highest ACE inhibitory activity (63.43%) compared with probiotic and yoghurt products. ▪ ACE inhibitory activity gradually increased during storage time.	[137]
Ultra-high temperature-treated milk	▪ ACE-inhibitory peptides such as VAPFPEVFGK, LVYPFPGPLH, and FVAPEPFVFGKEK	▪ ACE inhibitory activity	▪ VAPFPEVFGK, LVYPFPGPLH, and FVAPEPFVFGKEK, which are peptides from kombucha-fermented milk, exhibited high potency against ACE with IC_50_ values of 0.03, 0.03, and 0.75 mM, respectively.	[138]
*Eucalyptus camaldulensis* and *Litsea glaucescens*	▪ Phenolic compounds: gallocatechin, epigallocatechin, catechin, gallocatechin gallate, epicatechin, epigallocatechin gallate, epicatechin gallate ▪ Flavan-3-ols: rutin, kaempferol, quercetin	▪ ACE inhibitory activity	▪ *L. glaucescens* infusion had the highest ACE inhibitory activity (0.70 mg/mL), similar to captopril. ▪ Kombucha from *E. camaldulensis* and *L. glaucescens* kombuchas exhibit high potency against ACE with IC_50_ values of 2.61 and 1.08 mg/mL, respectively. ▪ Fermented with kombucha did not enhance ACE inhibitory potential of herbal infusions.	[140]

**Table 8:** Antihyperlipidemic effects of kombucha tea and kombucha beverages made from a variety of raw materials.

Name of Substrates	Active Ingredients	Biological Assays	Findings	Ref.
**In vivo studies**				
Green tea (*Camellia sinensis*)	▪ Phenolic compounds	▪ Wistar rats fed with a cholesterol-rich diet were given green tea (GT) or green tea kombucha (KT) at 5 mL per kg bw for 16 weeks. ▪ Plasma lipids (total cholesterol, triglyceride, LDL-C, VLDL-C, and HDL-C). ▪ Atherogenic index.	▪ GT significantly reduced TC, TG, LDL-C, and VLDL-C levels by 16, 22, 25, and 25%, respectively. ▪ KT significantly reduced TC, TG, LDL-C, and VLDL-C levels by 26, 27, 36, and 28%, respectively. ▪ GT and KT significantly increased HDL-C levels. ▪ GT and KT reduced the atherogenic index by 42 and 53%, respectively.	[51]
Traditional kombucha tea (TKT) and modified kombucha tea (MKT; tea broth fermented by a single *Gluconacetobacter* sp. A4)	▪ Phenolic compounds ▪ Organic acids: acetic acid, gluconic acid, ketogluconic acid	▪ Adult ICR mice fed with a hypercholesterolemic diet (HCD) were given TKT or MKT (66 mL/kg bw/day) for 12 weeks. ▪ Plasma lipids (TC, LDL-C, and HDL-C)	▪ MKT and TKT treatment lowered elevated TC levels to 82.21% and 82.03%, respectively, compared to HCD group. ▪ MKT and TKT treatment reduced LDL-C levels by 33.33, and 31.25%, respectively. ▪ The HDL-C levels were not altered in HCD mice treated with MKT and TKT.	[55]
Snake fruit (*Salak Suwaru* cultiva) and black tea	▪ Phenolic compounds	▪ Male Wistar rats were injected with streptozotocin to induce DM. DM rats were orally administered snake fruit kombucha (5 mL/kg bw/day) or metformin (45 mg/kg bw/day) for 28 days. ▪ Plasma lipids (TC, TG, and LDL-C).	▪ Snake fruit kombucha and black tea kombucha improved lipid profiles. ▪ Snake fruit kombucha has more effective in the improvement of lipid profiles than black tea kombucha.	[77]
Black tea (*Camellia sinensis*)	▪ Not determined	▪ Alloxan-induced diabetic rats were given black tea or kombucha tea at 5 mL per kg bw for 30 days. ▪ Plasma lipids (total cholesterol, triglyceride, and HDL-C). ▪ Plasma and pancreas lipase activity.	▪ Black tea and kombucha tea markedly reduced total cholesterol, triglyceride, and LDL-C levels. ▪ Black tea and kombucha tea significantly increased HDL-C level. ▪ Compared with black tea, kombucha tea was a better inhibitor of lipase activities in the plasma and pancreas.	[129]
Black tea (*Camellia sinensis*) and skim milk	▪ Not determined	▪ Adult male Wistar rats were treated with kombucha black tea or milk fermented by kombucha (3.6 mL/200 g bw/day) for 21 days. ▪ Plasma lipids (TG, LDL-C, VLDL-C, and HDL-C).	▪ Black tea kombucha and skim milk fermented with black tea kombucha reduced TG, VLDL-C, and LDL-C levels. ▪ Black tea kombucha and skim milk fermented with black tea kombucha increased HDL-C level. ▪ Skim milk fermented with black tea kombucha exhibited a higher efficacy compared with black tea kombucha.	[146]

## Data Availability

Data will be made available on request.

## Supplementary Materials

The following supporting information can be downloaded at https://www.mdpi.com/article/10.3390/foods12091818/s1: Table S1: Antioxidant activities of kombucha tea in in vitro studies.

## Abbreviations

AAB, acetic acid bacteria; AB, antibody; ABTS, 2,2′-azinobis-(3-ethylbenzotthiazoline-6-sulfonic acid; Ang II, angiotensin II; ACE, angiotensin-converting enzyme; CVD, cardiovascular disease; CAT, catalase; CHD, coronary heart disease; CUPRAC, cupric reducing antioxidant capacity; DCFH-DA, 2,7-dichlorofluorescein diacetate; DPPH, 2,2-diphenyl-1-picrylhydrazyl; DTH, delayed-type hypersensitivity; EC, epicatechin; EGC, epigallocatechin; EGCG, epigallocatechin-3-gallate; FRAP, ferric reducing antioxidant power; GCG, epicatechin-3-gallate; GPx, glutathione peroxidase; GRe, glutathione reductase; GSH, reduced glutathione; GST, glutathione-S-transferase; HEK-293, human embryonic kidney-293 cells; HO-1, heme oxygenase-1; HDL, high-density lipoprotein; LDL, low-density lipoprotein; MDA, malondialdehyde; Mn-SOD, manganese superoxide dismutase; NO, nitric oxide; ORAC, oxygen radical absorbance capacity; RNS, reactive nitrogen species; ROS, reactive oxygen species; SCOBY, symbiotic culture of bacteria and yeasts; SOD, superoxide dismutase; TBARS, thiobarbituric acid reactive substances; TC, total cholesterol; TFC, total flavonoid content; TG, triglyceride; TOAC, total antioxidant capacity; TPC, total phenolic content
