# Supplementary material for: Kombucha Beverages Produced from Fruits, Vegetables, and Plants: A Review on Their Pharmacological Activities and Health Benefits
Source: Foods. 2023 Apr 27;12(9):1818. doi: 10.3390/foods12091818 (PMC10178031; doi:10.3390/foods12091818)
Supplement: Supplementary file 1 [file foods-12-01818-s001.zip › foods-2357472-supplementary.pdf]

Supplementary Table S1: Antioxidant activities of kombucha tea

Name of fruits and vegetables	Active ingredients	Biological assays	Findings	Ref.
**In vitro studies**				
■ Black tea	■ Polyphenols: theaflavin, thearubigin, caffeine	■ DPPH, ABTS, and hydroxyl radicals scavenging assay<br>■ NO scavenging assay<br>■ FRAP assay	■ The free radical scavenging ability of kombucha tea had highest effect on the 7th day of fermentation and showed the decrease in IC_{50} values by 38.4% to 45.8% on the 21st day.<br>■ The reducing power of kombucha tea significantly increased by 35% and 44% in the 14th and 21st day of fermentation, respectively.	[18]
■ Black Ceylon, green, white, and red teas	■ Phenolic compounds<br>■ Flavonoids	■ DPPH radical scavenging assay<br>■ FRAP assay	■ Green tea kombucha showed the highest antioxidant property when compared with kombuchas made from other teas.<br>■ The highest DPPH scavenging activity of all kombuchas was observed at the 1st day of fermentation, then it became lower with the time of fermentation.<br>■ FRAP of the kombuchas increased after fermentation for 7 days. However, it decreased when observed at 14th day of fermentation.	[43]
■ Black tea	■ Polyphenols: theaflavin, thearubigin<br>■ Organic acids: acetic acid, gluconic acid, D-saccharic acid 1,4-lactone	■ DPPH, hydroxyl, and superoxide radicals scavenging assay	■ The free radical scavenging activities of kombucha on DPPH, hydroxyl and superoxide radicals increased by 18.9%, 17.2% and 14.97%, respectively.	[46]
■ Black tea	■ Phenolic compounds: gallic acid, catechin, (-)-epicatechin, caffeine, P-coumaric acid, ferulic acid, rutin hydrate, taxifolin, trans-cinnamic acid, 3-tert-butyl-4-hydroxybenzoic acid	■ DPPH scavenging assay	■ DPPH scavenging activity was associated with the phenolic content in the samples.<br>■ The ethyl acetate fractions showed highest inhibitory effect against DPPH with IC_{50} value of 9 µg/mL.<br>■ Whereas, the aqueous fractions had low activity with IC_{50} value of >50 µg/mL.	[47]
■ Black tea	■ Phenolic compounds	■ Copper (Cu^{2+})-induced lipid peroxidation inhibitory effect<br>■ DPPH, hydroxyl, and superoxide radicals scavenging assay	■ DPPH, hydroxyl, and superoxide radicals scavenging activities of traditional and modified kombuchas (fermented with *Gluconacetobacter* sp.) teas were found to increase during the fermentation process.<br>■ The kombucha samples could inhibit lipid peroxidation by prolonging the lag phase.	[48]
■ Black and green teas	■ Phenolic compounds: theaflavin, thearubigin, Catechin, callocatechin 3-*O*-gallate, epigallocatechin 3-*O*-gallate, catechin 3-*O*-gallate, catechin 5-*O*-gallate, quercetin glycosides 5-*O*-galloyquinic acid, 4-coumaroylquinic acid derivatives, caffeoylquinic acid derivatives	■ ABTS radicals scavenging assay	■ Black tea kombucha (13.59 µmol TE/mL) showed higher antioxidant capacity than green tea kombucha (8.22 µmol TE/mL).	[49]
■ Black and green teas	■ Phenolic compounds	■ DPPH, hydroxyl, and superoxide radicals scavenging assay<br>■ FRAP assay	■ Green tea kombucha showed higher scavenging abilities against DPPH, hydroxyl, and superoxide radicals than black tea kombucha.<br>■ Black tea kombucha had higher FRAP than green tea kombucha.	[50]
■ Green tea	■ Phenolic compounds	■ DPPH and superoxide radicals scavenging assay	■ Green tea kombucha had higher scavenging activity against DPPH radicals and superoxide anion than unfermented green tea.	[51]
■ Black, green, and oolong teas	■ Phenolic compounds<br>■ Organic acids: acetic acid, gluconic acid, D-saccharic acid 1,4-lactone, glucuronic acid, ascorbic acid	■ DPPH scavenging assay	■ DPPH scavenging activity of green, oolong, and black tea kombuchas was significantly elevated after 3 days of fermentation and the ability was increased up to 2.642, 2.582, and 0.435 mg GAE/mL at 15th, 9th and 3rd day of fermentation, respectively.	[52]

■ Chinese black (CBT), oolong (COT), green teas (CGT), and Sri Lankan black tea (SLBT)	■ Phenolic compounds: epicatechin isomers: (-)-epicatechin (EC), (-)-epicatechin-3-gallate (ECG), (-)-epigallocatechin (EGC), (-)-epigallocatechin-3-gallate (EGCG)	■ DPPH radical scavenging assay<br>■ ORAC assay	■ SLBT kombucha showed the highest DPPH radical scavenging and ORAC activities.<br>■ ORAC of SLBT and CGT kombuchas showed significant increase in 1st day of fermentation and then slightly change until 7th day of fermentation.	[53]
